# Enhanced Benzene Adsorption in Chloro-Functionalized Metal–Organic Frameworks

**DOI:** 10.1021/jacs.4c07207

**Published:** 2024-10-04

**Authors:** Yu Han, David Brooks, Meng He, Yinlin Chen, Wenyuan Huang, Boya Tang, Bing An, Xue Han, Meredydd Kippax-Jones, Mark D. Frogley, Sarah J. Day, Stephen P. Thompson, Svemir Rudić, Yongqiang Cheng, Luke L. Daemen, Anibal J. Ramirez-Cuesta, Catherine Dejoie, Martin Schröder, Sihai Yang

**Affiliations:** † Department of Chemistry, University of Manchester, Manchester M13 9PL, U.K.; ‡ College of Chemistry and Molecular Engineering, Beijing National Laboratory for Molecular Sciences, Peking University, Beijing 100871, China; § College of Chemistry, Beijing Normal University, Beijing 100875, China; ∥ Diamond Light Source, Harwell Science Campus, Oxfordshire OX11 0DE, U.K.; ⊥ ISIS Facility, Science and Technology Facilities Council, Rutherford Appleton Laboratory, Chilton OX11 0QX, U.K.; # Chemical and Engineering Materials Division (CEMD), Neutron Sciences Directorate, Oak Ridge National Laboratory, Oak Ridge, Tennessee 37831, United States; ∇ The European Synchrotron Radiation Facility, Beamline ID22, 71 Avenue des Martyrs, CS40220, Grenoble Cedex 9 38043, France

## Abstract

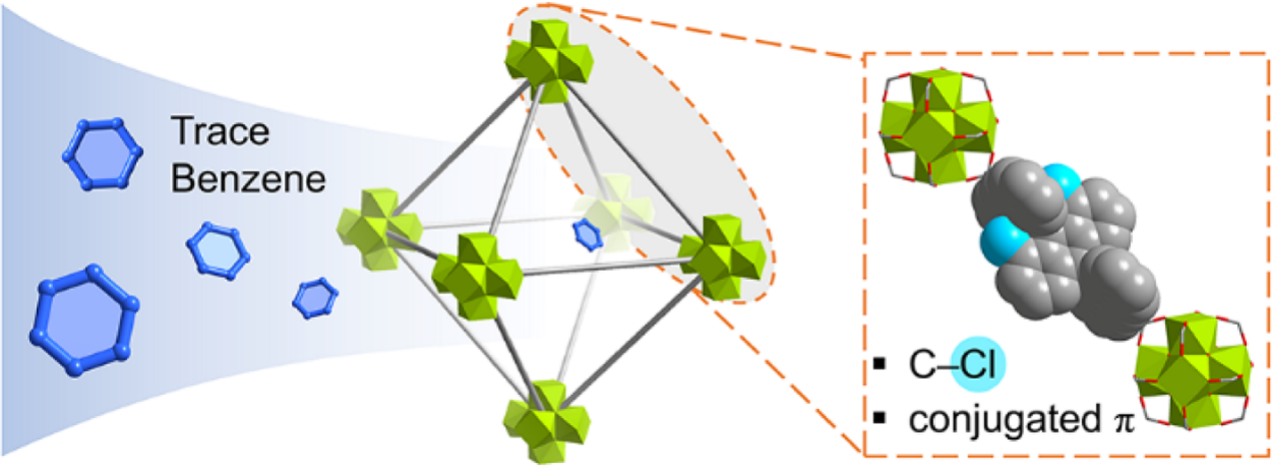

The functionalization of metal–organic frameworks (MOFs) to enhance the adsorption of benzene at trace levels remains a significant challenge. Here, we report the exceptional adsorption of trace benzene in a series of zirconium-based MOFs functionalized with chloro groups. Notably, MFM-68-Cl_2_, constructed from an anthracene linker incorporating chloro groups, exhibits a remarkable benzene uptake of 4.62 mmol g^–1^ at 298 K and 0.12 mbar, superior to benchmark materials. In situ synchrotron X-ray diffraction, Fourier transform infrared microspectroscopy, and inelastic neutron scattering, coupled with density functional theory modeling, reveal the mechanism of binding of benzene in these materials. Overall, the excellent adsorption performance is promoted by an unprecedented cooperation between chloro-groups, the optimized pore size, aromatic functionality, and the flexibility of the linkers in response to benzene uptake in MFM-68-Cl_2_. This study represents the first example of enhanced adsorption of trace benzene promoted by −CH···Cl and Cl···π interactions in porous materials.

## Introduction

Air pollution is linked to ca. 7 million premature deaths annually, as suggested by the World Health Organization (WHO).^1^ In particular, long-term exposure to trace-level benzene in indoor environments poses serious health risks due to its genotoxicity and carcinogenicity, with no safe level of exposure recommended by the WHO.^2−4^ Additionally, benzene also presents challenges in the production of cyclohexane, an important industrial process for the synthesis of nylon, solvents, and adipic acid.^5,6^ The presence of benzene residues in cyclohexane is harmful to product quality, environmental safety, and regulatory compliance. Therefore, developing effective strategies for capturing trace quantities of benzene and separating benzene from cyclohexane is of great importance. Sorption-based removal of benzene using porous materials has attracted much interest due to the ease of regeneration and the high potential adsorption capacity.^7−10^ Metal–organic framework (MOF) materials, with tunability and high internal surface areas, have demonstrated promise for adsorption of benzene, with, for example, MIL-101 showing an uptake of 16.7 mmol g^–1^ at 303 K and 80 mbar, and MOF-177 showing 16.8 mmol g^–1^ at 298 K and *P*/*P*_0_ = 1.^11−14^ However, adsorption of benzene at low concentrations is predominantly governed by the attractive forces of MOFs toward benzene and remains a challenge.^15−22^ Confined voids, often decorated with abundant aromatic sites, enable the binding of benzene through π···π interactions, which has been demonstrated in MFM-300(Sc) (3.02 mmol g^–1^ at 298 K and 1.2 mbar), ZJU-520(Al) (5.98 mmol g^–1^ at 298 K and 1.2 mbar), and BUT-55 (3.28 mmol g^–1^ at 298 K and 7.3 Pa).^23−27^ The constriction of pores resulting from a bulkier cubane-based ligand also promotes the formation of CH···π interactions for benzene adsorption in CUB-5 (7.6 mmol g^–1^ at 298 K and *P*/*P*_0_ = 0.11).^28−30^ Recently, open Cu(II) sites have been reported to afford specific binding affinity toward benzene via Cu(II)···π interactions, promoting an adsorption of benzene within UiO-66-Cu(II) of 3.92 mmol g^–1^ at 298 K and 1.2 mbar.^25^ Moreover, polar moieties in MOFs such as bridging μ_2_–OH and fluoro groups can provide hydrogen bonding interactions (−OH···π, −CH···F) to stabilize adsorbed benzene molecules.^24,31^ To date, tailoring the pore environment of MOFs to achieve efficient benzene removal at low pressures (*P*/*P*_0_ < 0.001) remains a significant challenge.

**Figure 1:**
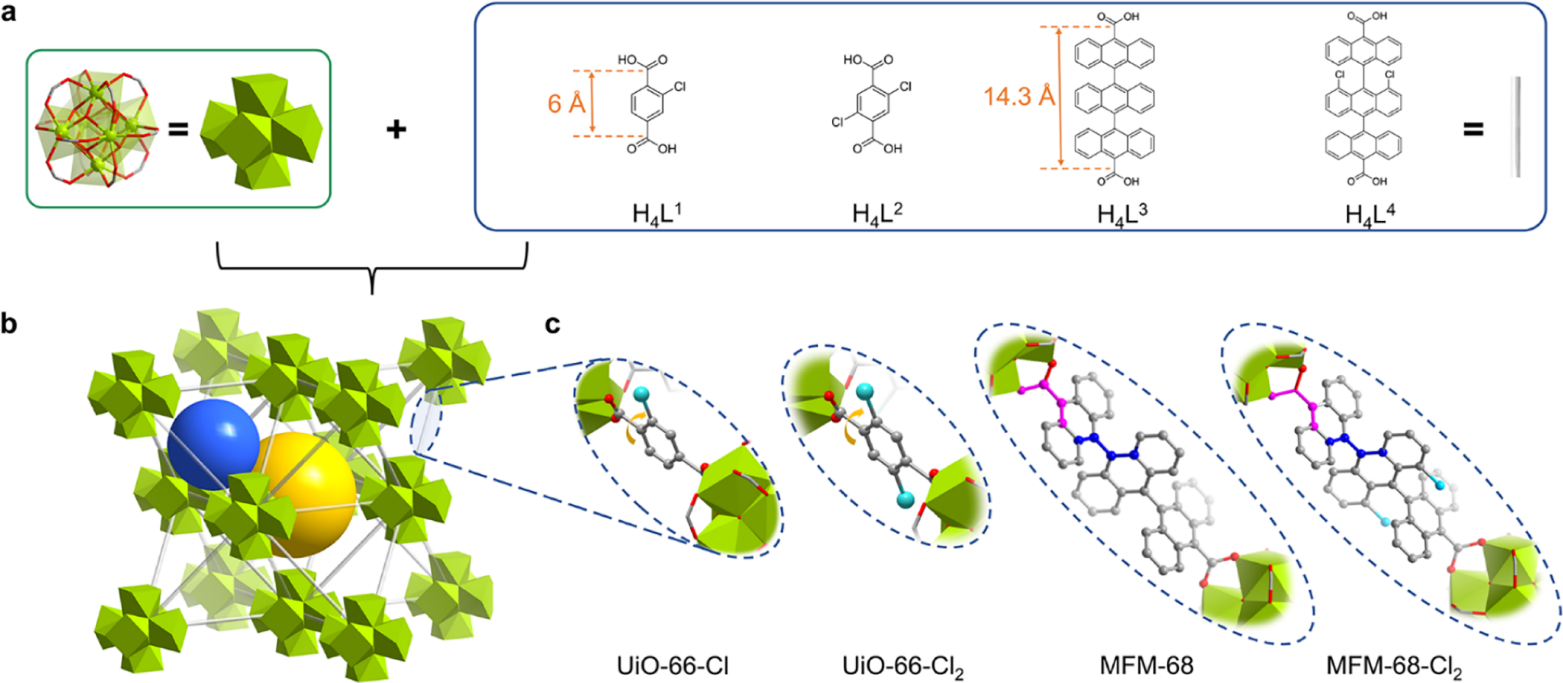
Schematic representation of the MOFs in this study. Illustration of (a) {Zr_6_} clusters and organic linkers and (b) *fcu* network topology. (c) Views of the local geometry of organic linkers. Ligands H_4_L^1^, H_4_L^2^, H_4_L^3^, and H_4_L^4^ correspond to UiO-66-Cl, UiO-66-Cl_2_, MFM-68, and MFM-68-Cl_2_, respectively. Hydrogen atoms are omitted for clarity.

Here, we report enhanced benzene adsorption in a series of Zr(IV)-based MOFs featuring chloro-functionalized linkers. Mono- and dichlorinated terephthalic acids, 2-chloroterephthalic acid (H_2_BDC-Cl) and 2,5-dichloroterephthalic acid (H_2_BDC-Cl_2_), have been used to synthesize two UiO-66 derivatives, UiO-66-Cl and UiO-66-Cl_2_, respectively. Chlorination of the linker increases benzene uptake from 0.63 mmol g^–1^ (in UiO-66^24^) to 0.88 mmol g^–1^ (in UiO-66-Cl) and 1.43 mmol g^–1^ (in UiO-66-Cl_2_) at 0.12 mbar and 298 K. However, the substitution of −H with −Cl on the linker inevitably reduces the accessible pore volume. To further enhance the uptake at low pressures, elongated linkers [9,9′:10′,9″-teranthracene]-10,10″-dicarboxylic acid (H_2_Teran) and its 1′,8′-dichlorinated derivative (H_2_Teran-Cl_2_) were used to construct two novel Zr-based MOFs, MFM-68 and MFM-68-Cl_2_, respectively (Figure 1). In addition to the polar −Cl group,^32−34^ conjugated anthracene moieties in Teran-Cl_2_ provide delocalized π-electrons to establish π···π interactions with benzene molecules.^35^ Additionally, the corrugated pore interior can further enhance interactions with benzene.^36^ At 298 K and 0.12 mbar, MFM-68-Cl_2_ exhibits a remarkable benzene uptake of 4.62 mmol g^–1^, surpassing the benchmark of 3.41 mmol g^–1^ by BUT-55.^26^ Furthermore, MFM-68-Cl_2_ demonstrates excellent potential for benzene/cyclohexane separation, even in the presence of water. We also report the direct visualization of binding domains in these materials using high-resolution synchrotron X-ray powder diffraction (SXPD). Binding dynamics have also been investigated using in situ synchrotron Fourier transform infrared (FTIR) microspectroscopy and inelastic neutron scattering (INS), coupled with density functional theory (DFT) modeling. The chloro-functionalized MFM-68-Cl_2_, combining tailored pore chemistry and geometry, promotes the design of efficient sorbent materials with a superior performance for benzene adsorption at low pressure.

## Results and Discussion

### Synthesis and Structure

Solvothermal reactions of ZrCl_4_ and H_2_BDC-Cl or H_2_BDC-Cl_2_ in dimethylformamide (DMF) afforded UiO-66-Cl or UiO-66-Cl_2_, respectively. Powder diffraction analysis confirms that they crystallize in the same cubic group *Fm3̅m* as UiO-66 (Figure S1). The crystal structures of UiO-66-Cl and UiO-66-Cl_2_ were obtained by Rietveld refinement of the SXPD data. The structure comprises 12-connected [Zr_6_(μ_3_-O)_4_(μ_3_–OH)_4_] clusters bridged by two connected terephthalate ligands. Owing to the electrostatic repulsion of −Cl with the adjacent carboxylate oxygen, the phenyl and the carboxylate planes twist to give torsion angles of ca. 17° in UiO-66-Cl and 22° in UiO-66-Cl_2_ (Figure S2).

MFM-68 and MFM-68-Cl_2_ were synthesized by the reaction of ZrCl_4_ with H_2_Teran or H_2_Teran-Cl_2_, respectively, in DMF with acetic acid as a modulator. The powder X-ray diffraction patterns of MFM-68 and MFM-68-Cl_2_ confirm that they are isostructural to UiO-68 (Figure S1). The replacement of terphenyl with teranthracene introduces local steric interactions, which break the coplanarity of phenyl rings. Rietveld refinement of the SXPD data indicates that the anthracene moieties in both MOFs are staggered and nearly perpendicular to each other (Figure S2). Similarly, repulsion between the anthracene moiety and the adjacent carboxylate group affords a torsion angle along the C–C bridge of ca. 56°. As a result, the central anthracene moiety is orientated toward the pore at a dihedral angle of ca. 31° with respect to the carboxylate plane (Table S2). Scanning electron microscopy (SEM) and energy dispersive X-ray spectroscopy (EDS) elemental mapping confirm the octahedral-shaped morphology and uniform distribution of Cl within the MFM-68-Cl_2_ structure (Figures S3 and S4). Thermogravimetric analysis (TGA) (Figure S5) confirms thermal stability up to ∼350 °C for these materials, with the desolvated MOFs displaying Brunauer–Emmett–Teller (BET) surface areas of 908–1428 m^2^ g^–1^ (Figure S6). Analysis of the pore size distribution suggests that the pore diameters in MFM-68 and MFM-68-Cl_2_ are both around 8.0 and 9.3 Å, corresponding to the tetrahedral and octahedral pores, respectively (Figure S7). The stability of MFM-68-Cl_2_ in the presence of moisture was confirmed by the retention of crystallinity and porosity on immersion in water for 7 days (Figures S8 and S9).

### Benzene Adsorption Analysis

**Figure 2:**
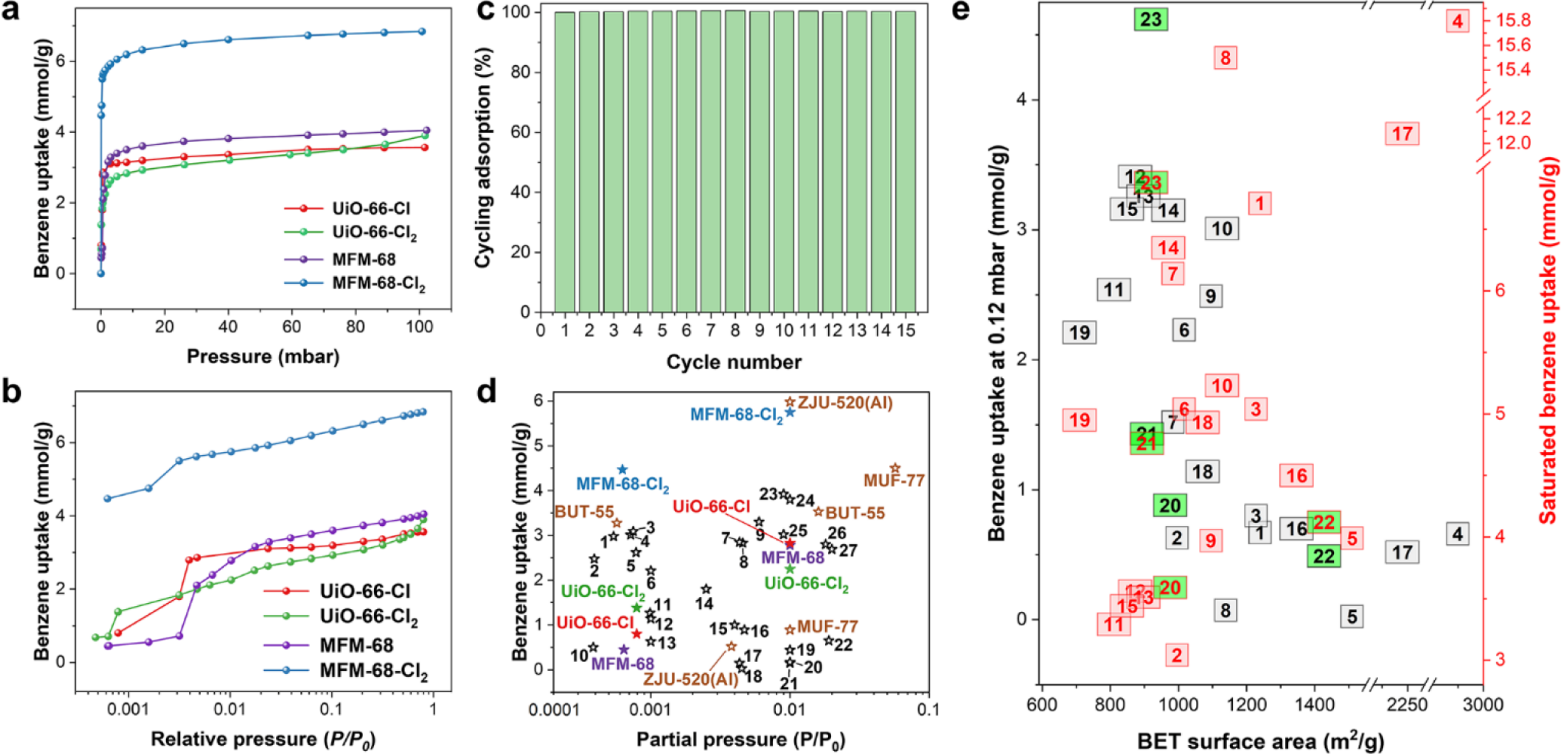
Benzene vapor adsorption data. (a) Benzene adsorption isotherms for UiO-66-Cl, UiO-66-Cl_2_, MFM-68, and MFM-68-Cl_2_ at 298 K (desorption data are shown in the Supporting Information). (b) Logarithmic-scale plots of *P*/*P*_0_ to view the adsorption of benzene at low partial pressures. (c) Cyclic adsorption–desorption of benzene in MFM-68-Cl_2_ at 298 K between 0 and 20 mbar. (d) Comparison of the low-pressure benzene uptakes of UiO-66-Cl, UiO-66-Cl_2_, MFM-68, and MFM-68-Cl_2_ with leading sorbents reported to date. Labels 1–27 refer to BUT-56^26^, BUT-53^26^, BUT-58^26^, BUT-57^26^, BUT-54^26^, STA-26-Et^28^, BUT-67^27^, BUT-66^27^, PAF-1^27^, MIL-101^27^, Zn(bdp) (bdp^2–^ = 1,4-benzenedipyrazolate)^26^, STA-26^28^, UiO-66^25^, Co(bdp)^26^, HKUST-1^26^, CUB-5^28^, MCM-41^27^, ZIF-8^27^, CUB-30^29^, BUT-12-Et^22^, BUT-12^22^, Zn(bpb) (bpb^2–^ = 1,2-bis(pyridine-2-carboxamido)benzenate)^26^, UiO-66-Cu(II)^25^, ZJU-620(Al)^24^, MFM-300(Sc)^25^, Ni(bpb)^26^, and MOF-177^26^, respectively. (e) Low-pressure benzene uptake (298 K, 0.12 mbar) and saturated benzene uptake (298 K, >100 mbar) vs Brunauer–Emmett–Teller (BET) surface area. Labels 1–23 refers to UiO-66-Cu(II)^25^, UiO-66^25^, MFM-300(Sc)^25^, MIL-101(Cr)^27^, ZIF-8^27^, Carboxen 1000^27^, BUT-67^27^, MCM-41^27^, BUT-66^27^, BUT-54^26^, BUT-53^26^, BUT-55^26^, BUT-56^26^, BUT-57^26^, BUT-58^26^, ZJU-620(Al)^24^, ZJU-520(Al), STA-26^28^, STA-26-Et^28^, UiO-66-Cl, UiO-66-Cl_2_, MFM-68, and MFM-68-Cl_2_, respectively.

The gravimetric adsorption isotherms for benzene vapor have been recorded for all four MOFs between 298 and 323 K (Figures 2a,b and S10). These isotherms exhibit steep increases at low pressures (*P*/*P*_0_ < 0.01) before reaching a plateau, indicating potential for the capture of benzene at trace levels. We evaluated the performance of these materials for benzene adsorption at low pressure (*P*/*P*_0_ < 0.001) (Table S3). At 298 K and 0.12 mbar, UiO-66-Cl and UiO-66-Cl_2_ show benzene uptakes of 0.88 and 1.43 mmol g^–1^, respectively, higher than that of pristine UiO-66 (0.63 mmol g^–1^).^24^ This demonstrates that the incorporation of −Cl functionalization can promote benzene adsorption at low pressure by up to 2.3-fold, representing the first example of Cl-enhanced benzene adsorption in MOFs. Significantly, MFM-68-Cl_2_ shows an exceptional benzene uptake of 4.62 mmol g^–1^ at 298 K and 0.12 mbar, exceeding the benchmark by BUT-55^26^ (3.41 mmol g^–1^) under the same conditions (Figure 2d). In contrast, pristine MFM-68 exhibits a lower uptake (by almost 10-fold) of 0.49 mmol g^–1^ at 298 K and 0.12 mbar, reflecting the absence of −Cl groups. The higher benzene uptake of MFM-68-Cl_2_ compared with that of UiO-66-Cl_2_ can be attributed to the larger pore volume in the former, providing more space to accommodate benzene molecules. Significantly, MFM-68-Cl_2_ exhibits no detectable reduction in either crystallinity or sorption capacity after 15 cycles of benzene adsorption–desorption (Figures 2c, S12, and S13). The isosteric heat of adsorption (*Q*_st_) for benzene uptake in MFM-68-Cl_2_ is 60–63 kJ mol^–1^ at a low surface coverage of 1.5–2.5 mmol g^–1^. This is higher than for MFM-68 (40–45 kJ mol^–1^) and suggests the presence of stronger host–guest interactions in MFM-68-Cl_2_ (Figure S14).

The correlation between benzene uptakes at low pressure (298 K and 0.12 mbar), saturated benzene uptakes (at 298 K), and BET surface area of state-of-the-art sorbents is presented in Figure 2e. As expected, higher surface areas afford higher saturated benzene uptakes but not necessarily higher uptakes in the low-pressure region. For example, MIL-101,^11,26^ with a high BET surface area of 2925 m^2^ g^–1^, exhibits a high saturated benzene uptake (15.8 mmol g^–1^ at 298 K and 120 mbar) but limited benzene uptake at low pressure (0.66 mmol g^–1^ at 298 K and 0.12 mbar). Indeed, the best-performing MOFs that exhibit high benzene adsorption at low pressure naturally show relatively low saturated benzene uptake, such as BUT-55^26^ (3.41 mmol g^–1^ at 298 K and 0.12 mbar, 3.62 mmol g^–1^ at 298 K and 123 mbar), BUT-56^26^ (3.26 mmol g^–1^ at 298 K and 0.12 mbar, 3.69 mmol g^–1^ at 298 K and 122 mbar), and BUT-66^27^ (2.49 mmol g^–1^ at 298 K and 0.12 mbar, 3.97 mmol g^–1^ at 298 K and 114 mbar). This reflects low pore space to accommodate guest benzene molecules at higher pressures, but confined nanopores can provide a strong affinity to benzene. Interestingly, MFM-68-Cl_2_ shows a high saturated benzene uptake of 6.88 mmol g^–1^ at 298 K and 100 mbar as well as an exceptional benzene uptake at 0.12 mbar. Thus, refining solely the pore size is insufficient to achieve the optimal benzene adsorption performance at both low and high pressures. In contrast, the synergistic tuning of pore chemistry (−Cl groups) and pore size reflects a balanced strategy for the development of benzene adsorbents.

To evaluate the performance of MFM-68-Cl_2_ in capturing trace benzene from ambient air, dynamic breakthrough experiments were performed with a gas mixture (5 ppm of benzene in air) flowed over a packed-bed of MFM-68-Cl_2_ under ambient conditions (Figure S15). Benzene was efficiently retained in the bed, with a 1% (0.05 ppm at outlet) breakthrough time of ∼60 000 min g^–1^, corresponding to a dynamic adsorption capacity of ∼2.68 mmol g^–1^. This surpasses the previously reported best value of 2.14 mmol g^–1^ for BUT-55 at 10 ppm of benzene.^26^ Additionally, water vapor adsorption experiments were conducted to assess the water adsorption behavior of MFM-68-Cl_2_ (Figure S16). Notably, MFM-68-Cl_2_ exhibits a quasi-linear isotherm with a low water uptake of 6.28 mmol g^–1^ at 298 K and *P*/*P*_0_ = 0.9, significantly lower than that of UiO-66-Cl_2_ (20.1 mmol g^–1^). The relative hydrophobicity of MFM-68-Cl_2_ can be attributed to the conjugated anthracene moieties on the pore surface. These results demonstrate the potential of MFM-68-Cl_2_ for benzene capture applications.

The separation of benzene and cyclohexane is an important but extremely challenging industrial process because of the small difference of 0.6 °C in their boiling points. The adsorption isotherms of cyclohexane were also measured for these four MOFs at 298–323 K (Figures S17 and S18). The lower uptakes and values of *Q*_st_ (Figure S14) for cyclohexane adsorption indicate a marked preference for benzene capture compared with that of cyclohexane. Specifically, MFM-68-Cl_2_ exhibits a record-high benzene/cyclohexane selectivity of 208 and 277 in liquid-phase and vapor-phase separation experiments, respectively (Figures S19 and S20). Additionally, benzene adsorption in MFM-68-Cl_2_ demonstrates rapid kinetics, reaching equilibrium within several minutes, faster than for cyclohexane adsorption (Figure S21). The adsorption of benzene and cyclohexane within these MOFs is highly reversible, and the adsorbed guest molecules can be fully removed by heating at 323 K under dynamic vacuum. PXRD patterns confirmed the retention of structures upon regeneration (Figure S22).

### Determination of Binding Domains

**Figure 3:**
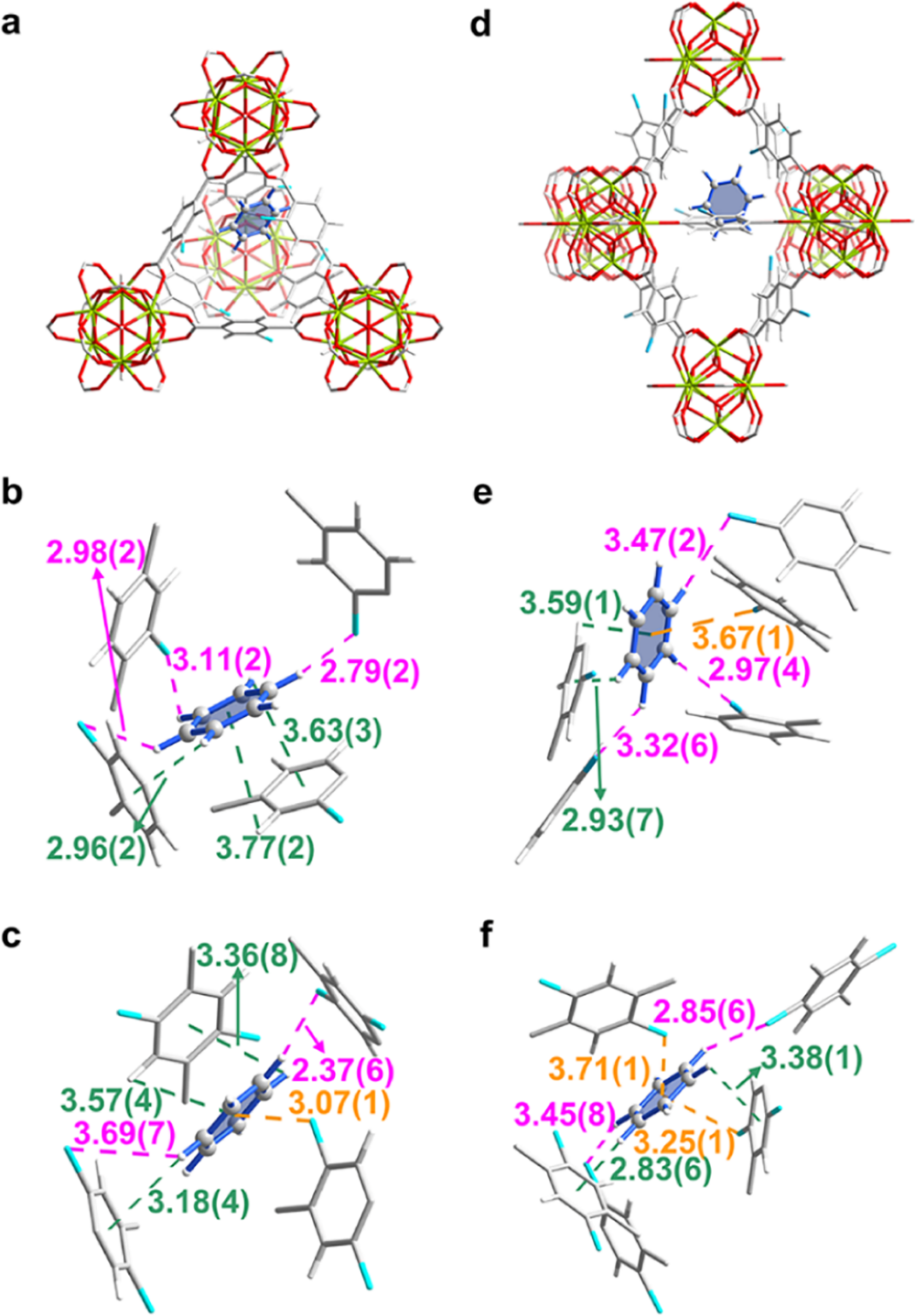
Views of the crystal structures of benzene-loaded UiO-66-Cl and UiO-66-Cl_2_ derived from Rietveld refinements of SXPD data. Views of binding site positions for benzene in the (a) tetrahedral and (d) octahedral cages in UiO-66-Cl. Views of (b) binding site I and (e) binding site II for benzene in UiO-66-Cl. Views of (c) site I and (f) site II for benzene in UiO-66-Cl_2_. Color code: Zr, lime; C, gray; O, red; H, white; Cl, blue.

**Figure 4:**
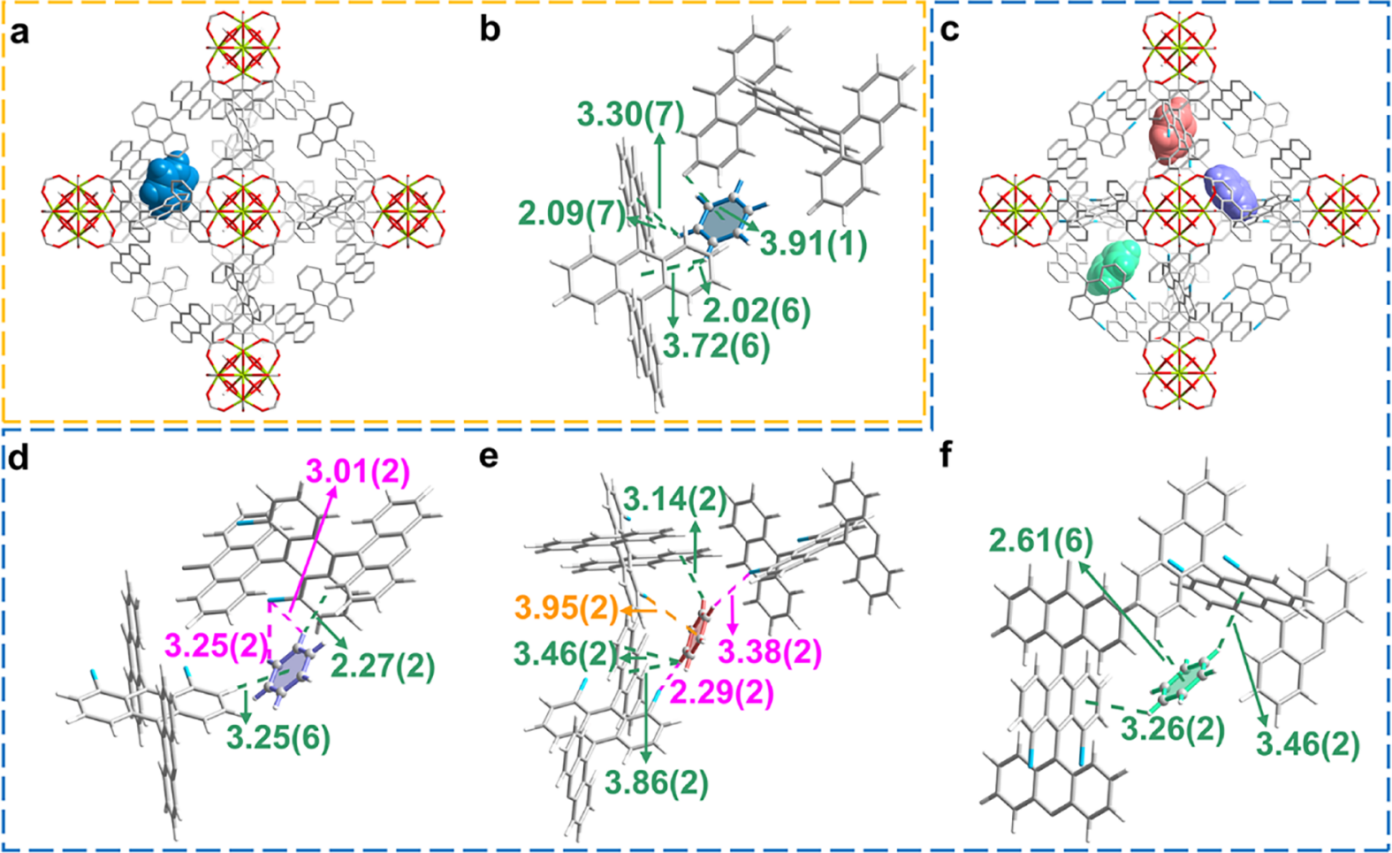
Views of the crystal structures of benzene-loaded MFM-68 and MFM-68-Cl_2_ derived from Rietveld refinement of SXPD data. Views of binding site positions for benzene in the octahedral cages of (a) MFM-68 and (c) MFM-68-Cl_2_ (hydrogen atoms are omitted for clarity). (b) Views of the single binding site for benzene in MFM-68. Views of (d) site I, (e) site II, and (f) site III for benzene in MFM-68-Cl_2_. Color code: Zr, lime; C, gray; O, red; H, white; Cl, blue.

High-resolution SXPD data for guest-loaded MOFs were collected at 298 K. Rietveld refinements of these data revealed distinct binding sites for both benzene and cyclohexane (Figures 3, 4, and S26–S31). Two binding sites for benzene, I and II, were found in UiO-66-Cl for benzene/{Zr_6_} = 3.91 and 2.14. Sites I and II are located in the tetrahedral and octahedral cavities, respectively (Figure 3a,d), with Site I interacting with ligand aromatic rings [−CH···π = 2.96(2)–3.77(2) Å] and −Cl groups [−CH···Cl = 2.79 (2)–3.11(2) Å] of the MOF (Figure 3b). Site II is stabilized by −CH···π interactions [distances of 2.93(7)–3.59(1) Å], −CH···Cl interactions [distances of 2.97 (4)–3.47(2) Å], and Cl···π interactions [distance of 3.67(1) Å] (Figure 3e). Similarly, two benzene sites are observed for UiO-66-Cl_2_. Site I (benzene/{Zr_6_} = 4.26) is within the tetrahedral cage, and site II (benzene/{Zr_6_} = 1.87) is located within the octahedral cage (Figure S26). Compared with UiO-66-Cl, both binding sites in UiO-66-Cl_2_ form strong interactions with −Cl [−CH···Cl = 2.37(6)–3.69(7) Å, Cl···π = 3.07(1)–3.71(1) Å] and framework phenyl rings [−CH···π = 2.83(6)–3.57(4) Å] (Figure 3c and f). This is coincident with the higher benzene uptake of UiO-66-Cl_2_ at low pressure compared to UiO-66-Cl, demonstrating the crucial role of −Cl groups in strengthening binding interactions with benzene. The presence of C ^δ+^–Cl^δ−^ dipoles can attract benzene via −CH···Cl hydrogen bonding and Cl···π halogen interactions. These types of interactions have been identified as important noncovalent contacts in biochemistry and materials science but have been rarely observed in host–guest interactions for benzene.^37−40^ Moreover, the chlorination of ligands can increase the −CH···π interactions between ligands and benzene.^35^ It is worth noting that as a response to host–guest interactions and local steric crowding, the chlorinated linkers reorientate along the diagonal *C*_2_ axis to form torsion angles of 17.6° and 22.6° with respect to the −COO plane of benzene-loaded UiO-66-Cl and UiO-66-Cl_2_, respectively (Table S4).

In benzene-loaded MFM-68, only one binding site (benzene/{Zr_6_} = 9.65) was observed and was located in the octahedral cavity. This is stabilized by −CH···π interactions between benzene and the aromatic rings of the ligand [distances of 2.02 (6)–3.91(1) Å] (Figure 4a,b). In contrast, there are three (I–III) distinct sites for benzene in MFM-68-Cl_2_ (Figure 4c). Site I and Site II (benzene/{Zr_6_} = 8.02 and 6.00, respectively) are anchored in the octahedral cage by −CH···π interactions [distances of 2.27(2)–3.86(2) Å], −CH···Cl interactions [distances of 2.29(2)–3.38(2) Å], and Cl···π interactions [distance of 3.95(2) Å] (Figure 4d,e). Site III (benzene/{Zr_6_} = 3.5) lies in the window connecting the tetrahedral and octahedral cages, and interacts with adjacent aromatic rings through −CH···π contacts [distances of 2.61(6)–3.46(2) Å] (Figure 4f). The additional Cl-induced binding interactions found in benzene-loaded MFM-68-Cl_2_ directly supports the observed exceptional adsorption of benzene. In addition, the enriched conjugated aromatic rings in MFM-68-Cl_2_, compared with UiO-66-Cl_2_, form stronger −CH···π interactions with benzene owing to the enhanced π-delocalization.^41,42^

The orientations of the teranthracene backbone of the linkers in MFM-68 and MFM-68-Cl_2_ respond differently upon benzene adsorption (Table S3). In MFM-68-Cl_2_, the central anthracene moiety flips away from the cage center with a reduction in torsion angles from 30.7° to 26.5°, in comparison to the −2.9° reduction in MFM-68. Simultaneously, the torsion angles between adjacent anthracene moieties are reduced from 93.1° to 82.6° in MFM-68-Cl_2_ and from 93.0° to 85.5° in MFM-68. However, the torsion angle between carboxylate and the first anthracene moiety shows little difference. Therefore, the rearrangement of linker conformations promotes the capture of benzene molecules. Furthermore, these perpendicularly staggered anthracene groups create undulating surfaces within the nanopores, which enhance host–guest interactions via overlapping van der Waals (vdW) potential surfaces that attract benzene.^34,36,43^ In contrast, all adsorbed cyclohexane molecules in these four MOFs bind weakly to the frameworks with −CH···C distances of 2.09(2)–3.02(7) Å with no π···π interactions. Furthermore, no notable interactions between the Cl groups and adsorbed cyclohexane molecules were observed, consistent with their poor adsorption.

### Analysis of Host–Guest Binding Dynamics

**Figure 5:**
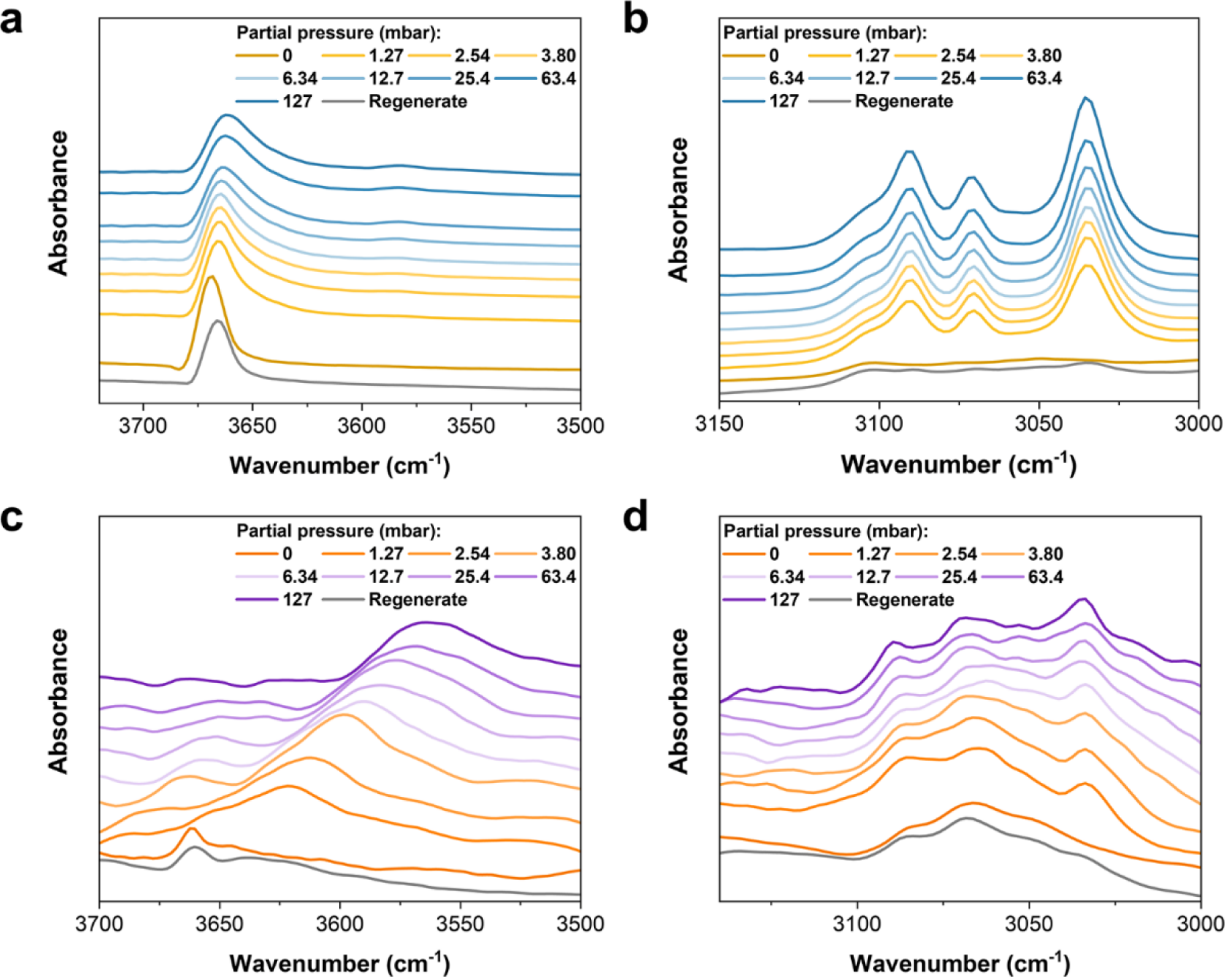
In situ FTIR spectra of UiO-66-Cl_2_ and MFM-68-Cl_2_. The (a,c) *v*(OH) and (b,d) CH stretching regions of (a,b) UiO-66-Cl_2_ and (c,d) MFM-68-Cl_2_ at partial pressures of benzene from 0–127 mbar (diluted in dry N_2_) at 298 K and after regeneration at 353 K with a flow of dry N_2_.

The in situ FTIR microspectroscopic studies of UiO-66-Cl_2_ and MFM-68-Cl_2_ as a function of benzene loading reveal a depletion of the bare O–H stretching bands at 3669 and 3661 cm^–1^, respectively, and the rapid emergence of red-shifted (Δ = 8–98 cm^–1^) and broadened O–H bands, indicating the interaction between benzene and the −OH moiety in the framework (Figure 5a,c).^44^ The red shifts suggests moderate interactions, consistent with the long distances observed between benzene and −OH groups in the structural analysis.^45^ In addition, the perturbation of the framework C–H band and the substantial growth of the benzene C–H band (3090 cm^–1^) demonstrate the interactions between benzene and aromatic rings of the framework (Figure 5b,d). Remarkably, these changes are immediately apparent even at low pressure (<1.27 mbar), aligning with the sharp adsorption of trace benzene. Following regeneration at 353 K under a dry flow of N_2_, the spectra revert to their original state, confirming the reversible adsorption of benzene in these MOFs. In contrast, the spectra undergo minimal changes upon the adsorption of cyclohexane, indicating the absence of notable host–guest interactions, consistent with the observed low uptake of cyclohexane (Figure S32).

INS experiments and DFT calculations were conducted for bare and benzene-loaded UiO-66-Cl_2_ to gain further insights into the dynamics of the framework and the translational/rotational motion of the adsorbed benzene molecule (Figures S33 and S34). The bare MOF exhibits no obvious peaks in the lattice mode region (20–320 cm^–1^), indicating that the complex hydrogen bonding network formed between the –Cl and –H atoms of the ligand, significantly constrains rotation of the phenyl rings. Upon adsorption of benzene, the emergent new peak at 45 cm^–1^ signifies a novel transitional mode for the adsorbed benzene likely moving between cages, correlating with the observed reduction in the intensity of benzene-related peaks at 320–1600 cm^–1^. The lower rotational frequencies of benzene about the C_6_ axis and one of the C_2_ axes indicate an anisotropic interaction, which is also demonstrated by the suppression of the rotational mode of the framework.

## Conclusions

We have demonstrated the enhanced adsorption of trace benzene in a series of chloro-decorated MOFs, promoted by the −CH···Cl and Cl···π interactions in the pores. MFM-68-Cl_2_, featuring −Cl groups and conjugated anthracene moieties, forms corrugated pore surfaces that exhibit optimal binding sites and shape. At 298 K and 0.12 mbar, MFM-68-Cl_2_ shows an exceptional benzene uptake of 4.62 mmol g^–1^, representing a benchmark for a benzene sorbent. This study will inspire the future development of porous materials for challenging trace benzene removal.

## Supporting Information Available

Detailed synthesis and characterization, adsorption and separation, SXPD, in situ FTIR and INS experiments, DFT calculations, PXRD, TGA, additional gas adsorption and benzene/cyclohexane separation data, Rietveld refinement of SXPD patterns, crystallographic information and additional views of crystal structures, and additional in situ FTIR and INS spectra (PDF)
