# Supplementary material for: Enhanced Benzene Adsorption in Chloro-Functionalized Metal–Organic Frameworks
Source: J Am Chem Soc. 2024 Oct 4;146(41):28080–7. doi: 10.1021/jacs.4c07207 (PMC11488476; doi:10.1021/jacs.4c07207)
Supplement: Supplementary file 1 — ja4c07207_si_001.pdf [file ja4c07207_si_001.pdf]

SUPPLEMENTARY INFORMATION

Enhanced Benzene Adsorption in Chloro-Functionalised Metal-Organic Frameworks

Yu Han[1], David Brooks[1], Meng He[1], Yinlin Chen[1], Wenyuan Huang[2], Boya Tang[1], Bing An[1], Xue Han[3], Meredydd Kippax-Jones[1,4], Mark D. Frogley[4], Sarah J. Day[4], Stephen P. Thompson[4], Svemir Rudić[5], Yongqiang Cheng[6], Luke L. Daemen[6], Anibal J. Ramirez-Cuesta[6], Catherine Dejoie[7], Martin Schröder[1]* and Sihai Yang[1,2]*

[1]Department of Chemistry, University of Manchester, Manchester, M13 9PL, UK
M.Schroder@manchester.ac.uk
[2]College of Chemistry and Molecular Engineering, Beijing National Laboratory for Molecular Sciences, Peking University, Beijing 100871, China Sihai.Yang@pku.edu.cn
[3]College of Chemistry, Beijing Normal University, Beijing 100875, China
[4]Diamond Light Source, Harwell Science Campus, Oxfordshire, OX11 0DE, UK
[5]ISIS Facility, Science and Technology Facilities Council, Rutherford Appleton Laboratory, Chilton, OX11 0QX, UK
[6]Chemical and Engineering Materials Division (CEMD), Neutron Sciences Directorate, Oak Ridge National Laboratory, Oak Ridge, Tennessee, 37831, USA
[7]The European Synchrotron Radiation Facility, Beamline ID22, 71 Avenue des Martyrs, CS40220, 38043 Grenoble Cedex 9, France

Table of Contents

1. Experimental Section

    1.1. Synthesis and Characterisation

    1.2. Adsorption Isotherms and Benzene/Cyclohexane Separation

    1.3. Structure Determination Using High Resolution SXPD

    1.4. *In Situ* FTIR spectroscopy

    1.5. INS and DFT Calculations

2. PXRD Patterns

3. SEM images and EDS elemental mapping

4. TGA Curves

5. Gas Sorption and Benzene/Cyclohexane separation

6. Rietveld Refinement of SXPD Patterns

7. Crystallographic Data

8. Views of Crystal Structures

9. *In Situ* FTIR Spectra

10. INS Spectra

11. References

1. Experimental Section

1.1. Synthesis and Characterisation

All reagents were obtained from commercial suppliers and used without additional purification. Organic manipulations were conducted under Ar atmosphere, employing dry solvents unless specified otherwise. ^{1}H NMR and ^{13}C NMR spectra were measured on a Bruker AV400 or AV500 spectrometers. High-resolution electrospray mass spectra were measured on a Bruker Micro-TOF spectrometer with samples dissolved in MeOH using both positive and negative mode from m/z. ICP-OES analysis was performed on a Thermo Scientific iCAP 6300 Duo, and Fourier Transform Infrared Spectroscopy (FT-IR) measurements were carried out with a Thermo Scientific Nicolet iS5-IR spectrometer in ATR sampling mode. Powder X-ray diffraction (PXRD) data were collected in flat plate mode over the 2θ range 5−50° on an X'pert multipurpose diffractometer using Cu-Kα radiation (λ = 1.54056 Å, 40 kV/30 mA). Thermo-gravimetric analysis (TGA) was carried out on a SDTQ600 TA instrument under air flow with a heating rate of 10 °C min^{-1}. Scanning electron microscopy (SEM) and energy dispersive spectroscopy (EDS) were run on a Tescan SC Mira3 scanning electron microscope.

Synthesis of [9,9':10',9''-teranthracene]-10,10''-dicarboxylic acid (H_2Teran) and its 1', 8'-dichlorinated derivative (H_2Teran-Cl_2). The synthetic route to H_2Teran is described herein. H_2Teran-Cl_2 was synthesized using an equal molar quantity of 1,8-dichloroanthraquinone instead of anthraquinone. In the following synthesis scheme, the 'R' groups represent H for H_2Teran and Cl for H_2Teran-Cl_2, respectively.

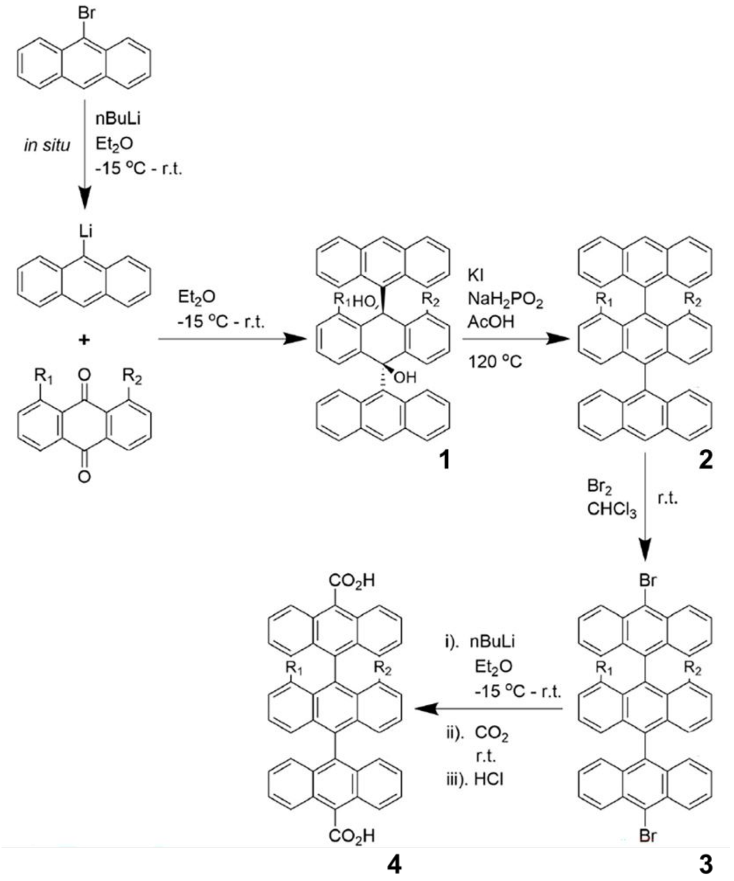

Preparation of [9,9':10',9''-teranthracene]-9',10'-diol (1). 9-Bromoanthracene (11.58 g, 45.0 mmol) in Et_2O (450 mL) was cooled to *ca.* -15 ˚C in a salted ice bath. n-Butyllithium (37.5 mL, 60.0 mmol, 1.6 M in hexane) was added cautiously over 30 mins and the orange suspension was stirred for a further 30 mins. Anthraquinone (3.12 g, 15.0 mmol) was added over 30 mins, the temperature elevated to room temperature and the mixture stirred for 16 h. The reaction was quenched with H_2O (300 mL), and the organic layer separated and washed with H_2O. The organic phase was dried over $MgSO_4$ and the solvent removed *in vacuo* to isolate the solid as a pale-yellow powder (5.80 g, 68%). ^{1}H NMR δ_H (DMSO-d$_6$, 400 MHz): 5.31 (2H, d, *J* = 2.1 Hz), 7.48 – 7.63 (12H, m), 8.15 (4H, d, *J* = 1.5 Hz), 8.20 – 8.31 (8H, m), 8.61 (2H, d, *J* = 2.2 Hz). ^{13}C NMR δ_C (100 MHz, DMSO-d$_6$): 78.41, 119.03, 127.40, 127.86, 129.55, 132.31, 144.77. MS (MALDI-ToF) *m/z*: 587.1 (M+Na)$^+$. IR (ATR) cm^{-1}: 3526 (O-H), 3030 (C-H, arom) 2970 (C-H, alkyl). Elemental analysis (% calcd/found): C 89.34/92.61, H 5.00/4.97.

Preparation of 9,9':10',9''-teranthracene (2). A mixture of **1** (3.39 g, 6.0 mmol), potassium iodide (9.0 g, 42.1 mmol), sodium hypophosphite (9.0 g, 102.2 mmol) and acetic acid (70 mL) was refluxed for 2 h under 120 ˚C. After cooling to room temperature, the precipitate was vacuum filtered and washed with H_2O and methanol to yield the product as a pale-yellow powder (3.03 g, 95%). Yellow needles were obtained by recrystallization from NMP. ^{1}H NMR δ_H ($CDCl_3$, 400 MHz): 7.40 – 7.49 (12H, m), 7.97 – 8.14 (12H, m), 8.50 (2H, d, *J* = 2.2 Hz). ^{13}C NMR δ_C (100 MHz, $CDCl_3$): 126.36, 126.90, 127.41, 128.17, 131.94, 134.27, 135.51. MS (MALDI-ToF) *m/z*: 530.2 (M)$^+$. IR (ATR) cm^{-1}: 3045 (C-H). Elemental analysis (% calcd/found): C 95.06/94.87 H 4.94/4.96.

Preparation of 10,10''-dibromo-9,9':10',9''-teranthracene (3). A solution of Br_2 (0.48 mL, 9.0 mmol) in $CHCl_3$ (60 mL) was added cautiously over 30 mins to a solution of **2** (1.59 g, 3.0 mmol) in $CHCl_3$ (150 mL) in air. The mixture was stirred at room temperature for 16 h and poured into an EtOH bath (300 mL) to induce precipitation. The solid was isolated by vacuum filtration and washed with copious EtOH to yield the product as a yellow powder (1.49 g, 72%). ^{1}H NMR δ_H ($CDCl_3$, 400 MHz): 7.61 – 7.75 (12H, m), 8.11 – 8.29 (12H, m). ^{13}C NMR δ_C (100 MHz, $CDCl_3$): 121.79, 125.22, 125.66, 126.09, 126.60, 127.94, 131.85, 134.06, 135.28. MS (MALDI-ToF) *m/z*: 688.5 (M)$^+$. IR (ATR) cm^{-1}: 3038 (C-H), 752 (C-Br). Elemental analysis (% calcd/found): C 73.27/77.14 H 3.51/3.98 Br 23.21/20.11.

Preparation of [9,9':10',9''-teranthracene]-10,10''-dicarboxylic acid (4). 3 (1.10 g, 1.6 mmol) in Et_2O (30 mL) was cooled to *ca.* -15 ˚C in a salted ice bath. *n*-Butyllithium (3.0 mL, 4.8 mmol, 1.6 M in hexane) was added cautiously over 30 mins and the orange suspension stirred for a further 30 mins. The temperature was elevated to room temperature and CO_2 bubbled through the mixture for 3 h, inducing a colour change to yellow. The reaction was diluted with Et_2O (20 mL) and quenched with H_2O (20 mL), causing precipitation of the product. The organic phase was removed *in vacuo* and the pH of the aqueous dispersion was adjusted to pH = 1 with 1 M H_2SO_4 to stimulate further precipitation. The solid was isolated as a pale yellow powder by vacuum filtration and washed with H_2O and Et_2O (0.72 g, 73%). ^{1}H NMR δ_H (DMSO-d$_6$, 400 MHz): 7.68 – 7.83 (12H,

m), 8.18 – 8.35 (12H, m). ^{13}C NMR δ_C (100 MHz, DMSO-d_6): 120.87, 124.85, 125.21, 125.60, 126.14, 126.89, 127.44, 127.95, 132.00, 134.55, 135.84. MS (MALDI-ToF) *m/z*: 618.2 $(M)^+$. IR (ATR) cm^{-1}: 3050 (C-H), 2569 (O-H), 1694 (C=O). Elemental analysis (% calcd/found): C 85.42/85.39 H 4.24/4.23.

Synthesis of MFM-68 and MFM-68-Cl$_2$. H_2Teran (61.9 mg, 0.1 mmol) and $ZrCl_4$ (23.3 mg, 0.1 mmol) were dissolved in a mixture of DMF (3.0 mL) and acetic acid (0.6 mL) in a 3-dram vial. The resulting mixture was heated at 120 ˚C for 24 h and then cooled slowly (*ca.* 2 ˚C min^{-1}) to room temperature. The product (MFM-68) was washed with DMF and diethyl ether then dried in air to afford pale yellow nanocrystallites (46.1 mg, 63%). IR (ATR) cm^{-1}: 3058 (C-H), 1652 (C=O). Elemental analysis (% calcd/found): C 72.47/72.89 H 3.32/3.40. MFM-68-Cl$_2$ was synthesized via the same procedure using equal molar quantity of H_2Teran-Cl$_2$ instead of H_2Teran.

Synthesis of UiO-66-Cl and UiO-66-Cl$_2$. UiO-66-Cl and UiO-66-Cl$_2$ were prepared according to the previously reported method with modifications.[1] 2-Chloroterephthalic acid (64.2 mg, 0.32 mmol), zirconium tetrachloride (34 mg, 0.15 mmol) and DMF (27 mL) were mixed together and transferred into a Teflon-lined stainless steel autoclave, which was sealed and heated at 120 °C for 24 h. After cooling to room temperature naturally, the resulting product was collected by centrifugation and washed with DMF and acetone for several times then dried in air to afford UiO-66-Cl. UiO-66-Cl$_2$ was synthesized *via* the same procedure using an equal molar quantity of 2, 5-dichloroterephathalic acid.

Characterisation of Porosity. The as-synthesised MOF samples were activated at 423 K and 10^{-10} bar for 12 h to afford the fully desolvated samples, which were loaded in a Micrometrics 3Flex analyser for porosity analysis. The BET surface areas were calculated using the N_2 isotherms measured at 77 K.

1.2. Gas adsorption and Benzene/Cyclohexane Separation

Vapor adsorption isotherms for benzene and cyclohexane were measured on an Intelligent Gravimetric Analyzer (IGA) (Hiden Isochema, Warrington, UK). MOF samples were loaded into the system and degassed at 423 K and 10^{-10} bar for 12 h to give fully desolvated samples. Benzene adsorption/desorption cycling experiments were conducted for MIL-125-Zn at 298 K and 0–20 mbar.

***Q_{st}* Calculation:** The isosteric enthalpy (ΔH_n) and entropy (ΔS_n) for the adsorption of benzene and cyclohexane were calculated as functions of loading (n). These calculations were based on isotherms measured between 298 K and 323 K, which were fitted using the van't Hoff isochore:

$$\ln(\mathrm{p})_n = \frac{\Delta H_n}{RT} - \frac{\Delta S_n}{R}$$

A plot of ln*(p)* versus $1/T$ at constant loading allows the differential enthalpy and entropy of adsorption and the isosteric enthalpy of adsorption (Q_{st}, n) to be determined.

Breakthrough experiments were conducted using a fixed-bed tube with a diameter of 3 mm and length of 50 mm, packed with approximately 10 mg of host material. The material was heated to 150 °C under a flow of

He overnight to ensure complete activation. On cooling the fixed bed to room temperature, the breakthrough experiment was performed using a calibration gas containing 5 ppm benzene balanced with air at atmospheric pressure and room temperature. The flow rate of the incoming gas was maintained at 200 mL min^{-1}, and the benzene concentration at the outlet was determined by mass spectrometry. The breakthrough curve was recorded until 1% of the initial concentration (i.e., 0.05 ppm) was detected in the eluted stream. This is higher than the detection limit of the mass spectrometer (< 0.01 ppm), and is recorded as 1% breakthrough time.

Liquid-phase benzene/cyclohexane separation experiments were carried out according to the previously reported method.[2,3] 50 mg of activated MOF adsorbent was immersed in 10 mL of a 1/1/0.1 (v/v/v) benzene/cyclohexane/water mixture for 24 h. After drying the MOFs in air for *ca.* 10 min to remove the solvent on the surface, the adsorbates in MOFs were extracted by DMSO-d_6 for ^{1}H NMR spectroscopic analysis. Vapor-phase separation experiments were conducted using a similar procedure, except that the activated MOF adsorbent was exposed to a vapor atmosphere in a sealed vial containing a 1/1/0.1 (v/v/v) benzene/cyclohexane/water mixture for 24 h.

1.3. Structure Determination Using High Resolution SXPD

High-resolution synchrotron powder X-ray diffraction (SXPD) patterns for benzene and cyclohexane loaded UiO-66-Cl/Cl_2 were collected at the beamline ID22 of European Synchrotron Radiation Facility (ESRF) using a multi-analyser crystal (MAC) detector with a monochromated X-ray radiation of λ = 0.35424 Å. For the bare MFM-68 and MFM-68-Cl_2, as well as C_6H_6- and C_6H_{12}-loaded MFM-68 and MFM-68-Cl_2, the SXPD patterns were collected at the beamline I11 of Diamond Light Source (DLS) using a set of 5 MAC detectors with an X-ray wavelength of λ = 0.825894 Å. The samples were loaded into 0.7 mm borosilicate capillary tubes and desolvated at 423 K and 10^{-10} bar for 12 h followed by exposure to saturated benzene or cyclohexane vapour at room temperature for at least 12 h prior to sealing. Diffraction patterns were collected at room temperature. Rietveld structure refinement on SXPD patterns were carried out using the TOPAS-Academic v5 package. Pawley refinement was first conducted to obtain the refined non-structural parameters, including background, peak shape, and lattice parameters. Structure models for the framework were established based on the crystal structure of UiO-66 and UiO-68. Structures of the organic linker were described using semi-rigid bodies where reasonable constraints were applied on the bond angle and distance. The phenyl ring in BDC^{2-} linker and the anthracene in Teran linker were allowed to freely rotate along the C_2 axis during the refinement. Rigid body model was used to define the molecular structure of benzene and cyclohexane (in chair conformation). The initial centre of masses and orientations of the rigid bodies were obtained using the simulated annealing method (the Auto_T() macro in Topas). At the final stage, all parameters were released to be refined and structure solutions were accepted with minimal agreement factors (R_{wp}).

1.4. *In Situ* FTIR spectroscopy

In situ Fourier transform infrared (FTIR) microspectroscopy was carried out at the Multimode InfraRed Imaging and Microspectroscopy (MIRIAM) beamline at the Diamond Light Source (UK). Spectra were collected (512 scans) over the range of 500–4,000 cm^{-1} at a resolution of 4 cm^{-1}, with an infrared spot size at

the sample of approximately 15 × 15 μm. Samples were placed onto a zinc selenide (ZnSe) disk and placed within a Linkam FTIR 600 gas-tight sample cell equipped with ZnSe windows, a heating stage, and gas inlet/outlets. A controlled mixture of benzene/cyclohexane vapor in N_2, generated by bubbling dry N_2 through liquid benzene/cyclohexane, along with pure dry N_2, was introduced into the sample cell at a regulated partial pressure, modulated by varying flow rates. Samples were desolvated under dry N_2 flow at 423 K for 2 h, cooled to 298 K, and dosed with benzene/cyclohexane vapor at different partial pressures (0–127 mbar for benzene, 0–130 mbar for cyclohexane).

1.5. INS and DFT Calculations

INS spectra were recorded on the VISION spectrometer at the Spallation Neutron Source, Oak Ridge National Laboratory (USA) and TOSCA Facility at Rutherford Appleton Laboratory (UK). MOF samples were desolvated at 423 K and 10^{-10} bar for 12 h followed by exposure to saturated benzene vapour at room temperature for at least 12 h. The powder was then transferred into cylindrical vanadium sample cells with an indium seal in the glovebox. The temperature during data collection was kept below 10 K.

Modelling by Density Functional Theory (DFT) of the bare and C_6H_6-loaded UiO-66-Cl_2 was performed using the Vienna Ab initio Simulation Package (VASP).[4] The calculation used Projector Augmented Wave (PAW) method[5,6] to describe the effects of core electrons, and Perdew-Burke-Ernzerhof (PBE)[7] implementation of the Generalized Gradient Approximation (GGA) for the exchange-correlation functional. Energy cutoff was 800eV for the plane-wave basis of the valence electrons. The lattice parameters and atomic coordinates determined in this work were used as the initial structure. Given the large size of the unit cell, the electronic structure was calculated on Γ-point only. The total energy tolerance for electronic energy minimization was 10^{-8} eV, and for structure optimization it was 10^{-7} eV. The maximum interatomic force after relaxation was below 0.002 eV/Å. The optB86b-vdW functional[8] for dispersion corrections was applied. The vibrational eigen-frequencies and modes were then calculated by solving the force constants and dynamical matrix using finite displacement method with Phonopy.[9] The OCLIMAX software[10] was used to convert the DFT-calculated phonon results to the simulated INS spectra.

2. PXRD Patterns

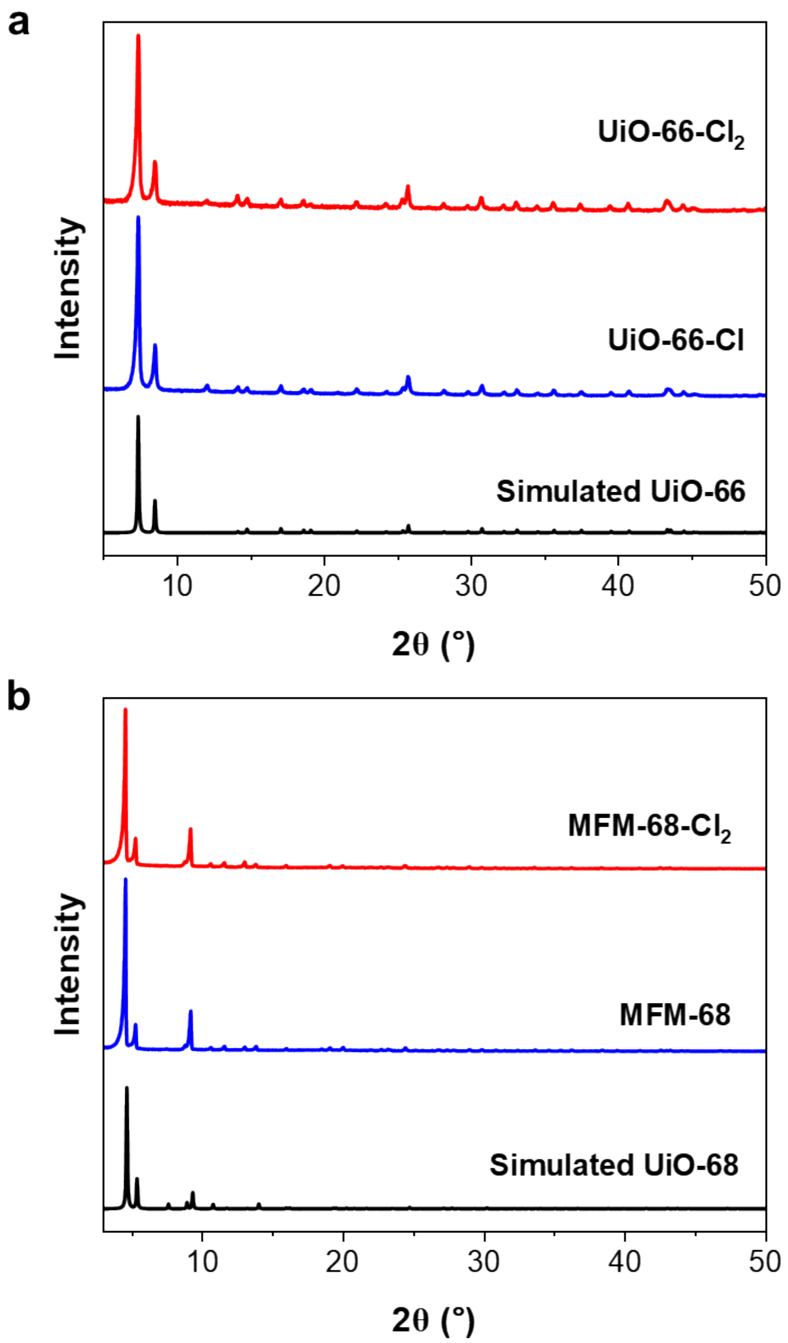

Figure S1. PXRD patterns for (a) UiO-66-Cl, UiO-66-Cl_2 and UiO-66, and of (b) MFM-68, MFM-68-Cl_2 and UiO-68.

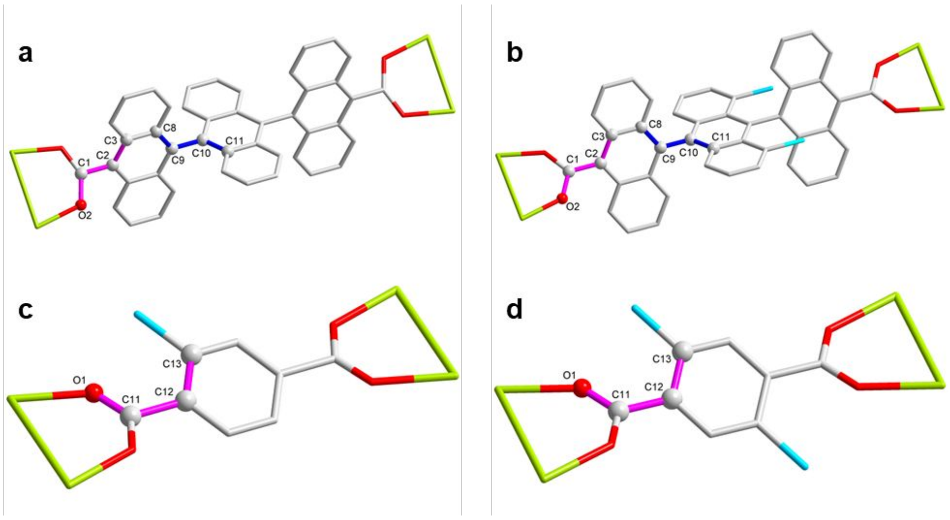

Figure S2. Local linker environment between {Zr_6} clusters. (a) MFM-68, (b) MFM-68-Cl_2, (c) UiO-66-Cl and (d) UiO-66-Cl_2. Colour code: Zr, lime; C, gray; O, red; Cl, blue. Hydrogen atoms are omitted for clarity.

Table S1. Torsion angles in linkers and between linker and Zr_6 cluster in MFM-68 and MFM-68-Cl_2.

Structure	Atom No.		
	C3–C2–C1–O2	C8–C9–C10–C11	O2–C1–C10–C11
MFM-68	56.0°	93.0°	30.9°
C_6H_6@MFM-68	57.5°	85.5°	28°
C_6H_{12}@MFM-68	54.2°	85.8°	35.3°
MFM-68-Cl_2	56.3°	93.1°	30.7°
C_6H_6@MFM-68-Cl_2	56.1°	82.6°	26.5°
C_6H_{12}@MFM-68-Cl_2	51.8°	78.9°	27.1°

Table S2. Torsion angles in linkers and between linker and Zr_6 cluster in UiO-66, UiO-66-Cl and UiO-66-Cl_2.

Structure	Atom No.
	O1–C11–C12–C13
UiO-66	0
C_6H_6@UiO-66-Cl	17.6°
C_6H_{12}@UiO-66-Cl	17.6°
C_6H_6@UiO-66-Cl_2	22.6°
C_6H_{12}@UiO-66-Cl_2	22.3°

3. SEM images and EDS elemental mapping

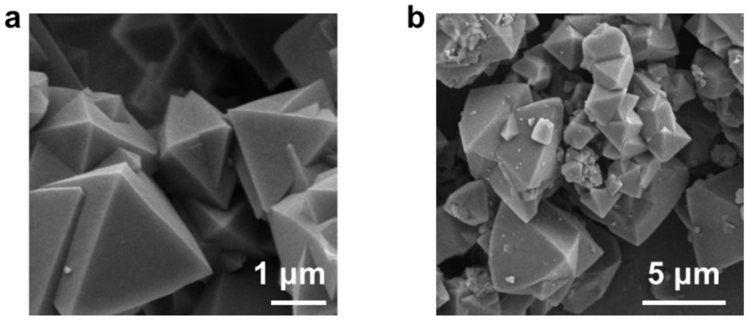

Figure S3. SEM images of (a) MFM-68 and (b) MFM-68-Cl_2.

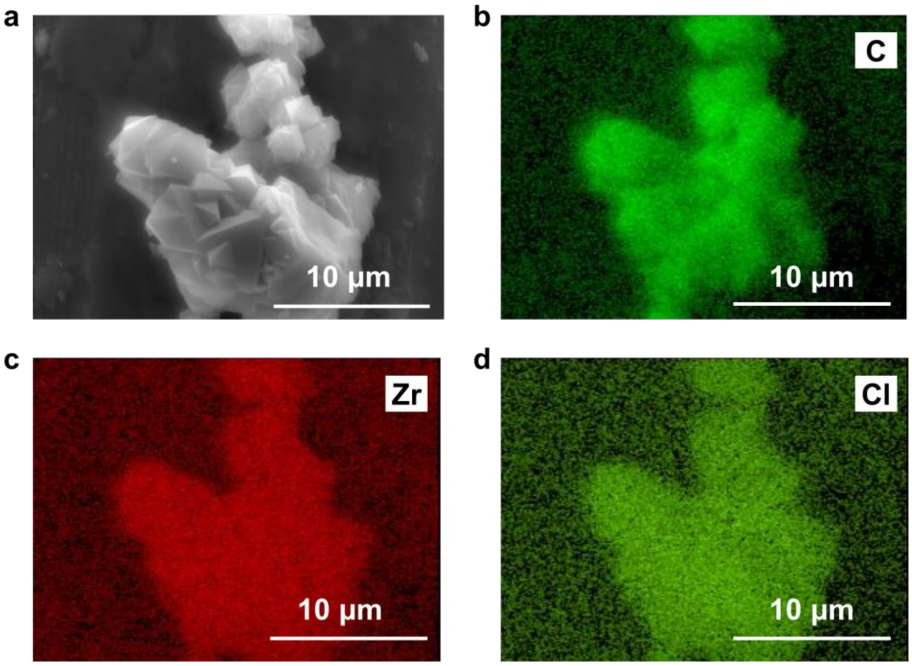

Figure S4. (a) SEM micrograph and (b-d) EDS elemental mapping of MFM-68-Cl_2.

4. TGA curves

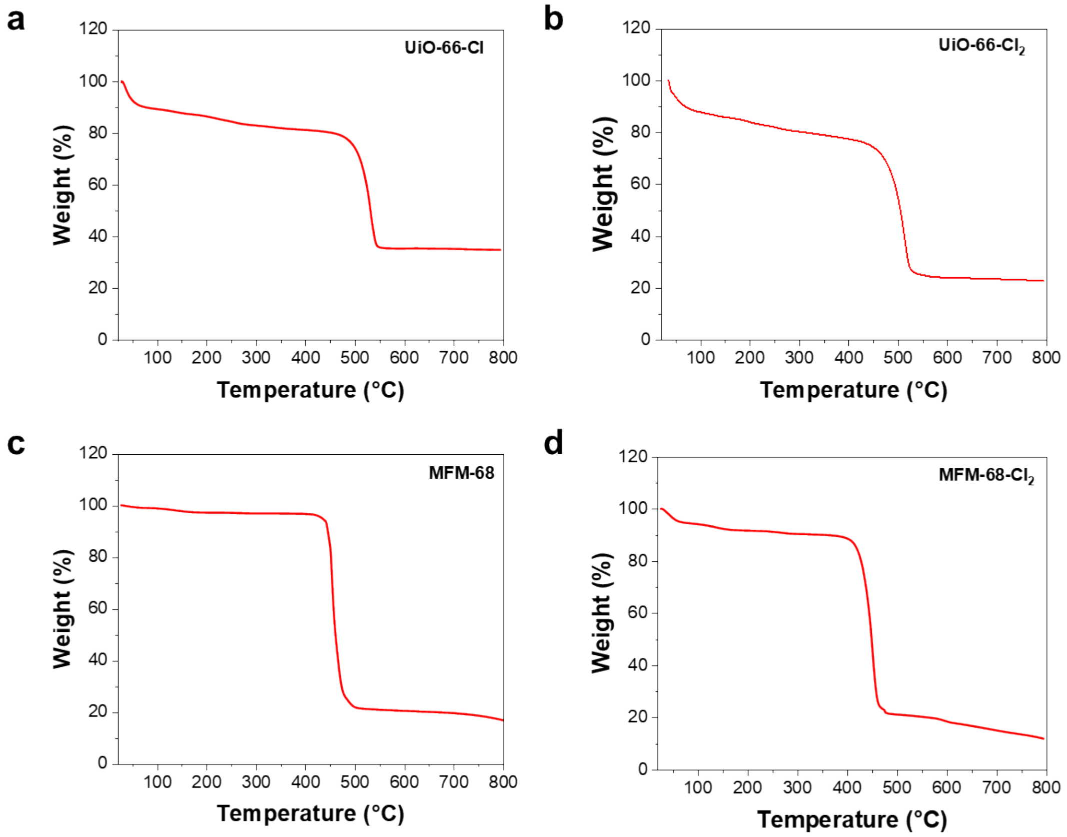

Figure S5. TGA curves of (a) UiO-66-Cl, (b) UiO-66-Cl$_2$, (c) MFM-68 and (d) MFM-68-Cl$_2$.

5. Gas Sorption and Benzene/Cyclohexane Separation

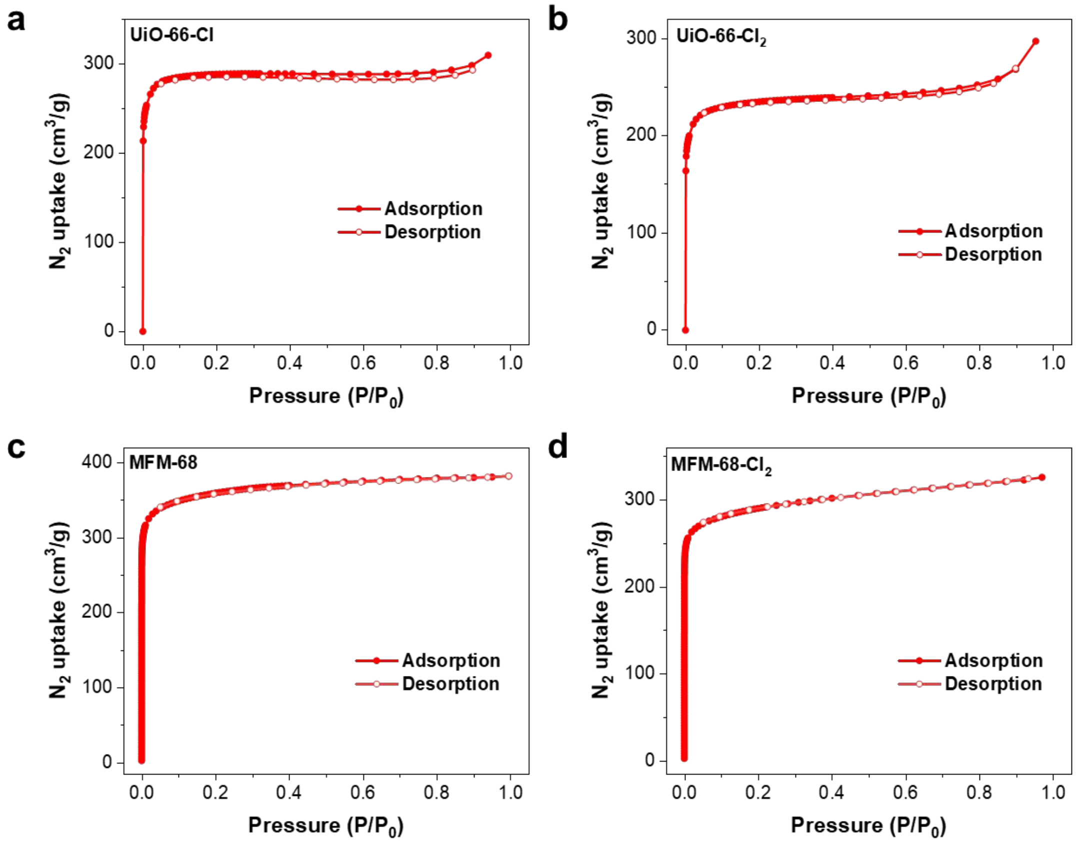

Figure S6. N_2 isotherms for (a) UiO-66-Cl, (b) UiO-66-Cl_2, (c) MFM-68 and (d) MFM-68-Cl_2 at 77 K (adsorption, solid symbols; desorption, open symbols).

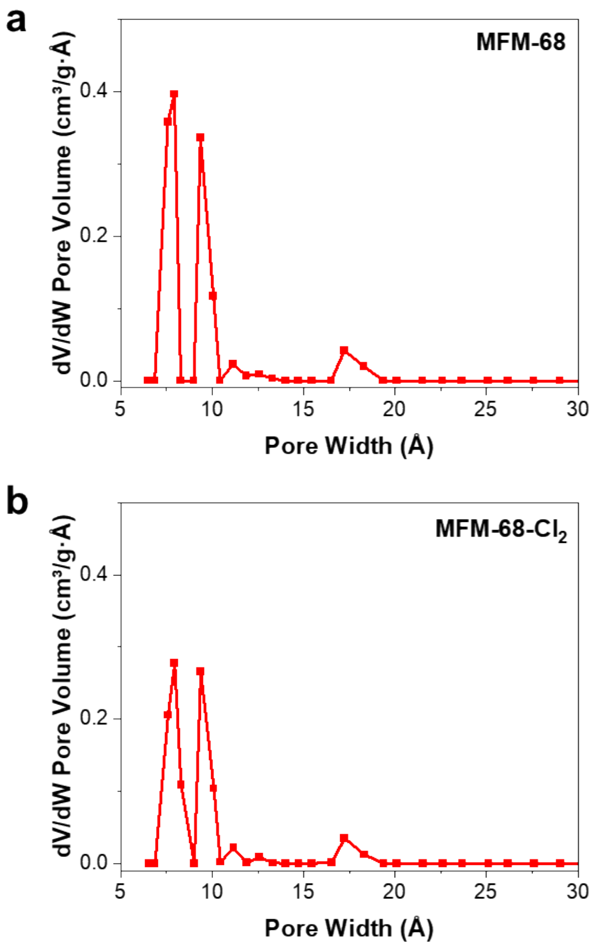

Figure S7. Pore size distribution of (a) MFM-68 and (b) MFM-68-Cl_2 determined by DFT derived from N_2 isotherms at 77 K. Peaks near 8.0 Å and 9.3 Å represent tetrahedral and octahedral pores, respectively.

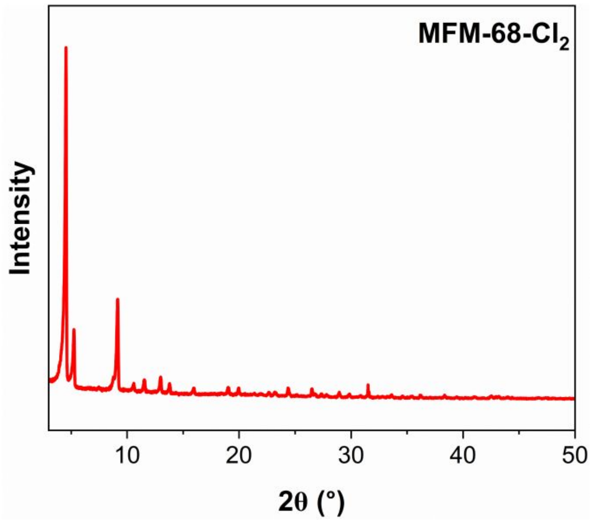

Figure S8. PXRD pattern for MFM-68-Cl_2 after being immersed in water for 7 days.

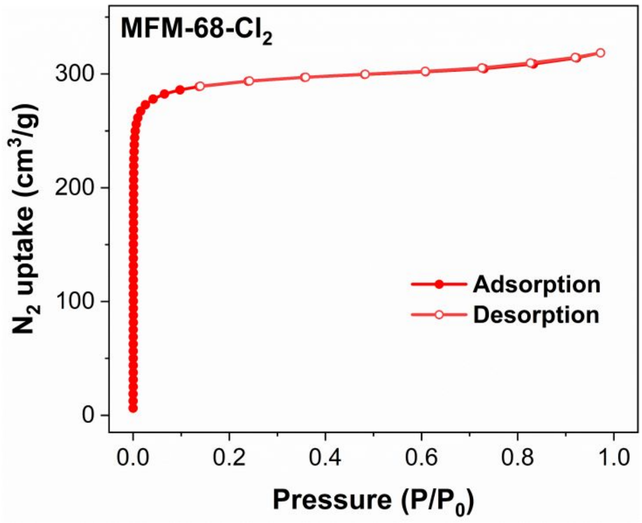

Figure S9. N_2 isotherms for MFM-68-Cl_2 at 77 K after being immersed in water for 7 days (adsorption, solid symbols; desorption, open symbols).

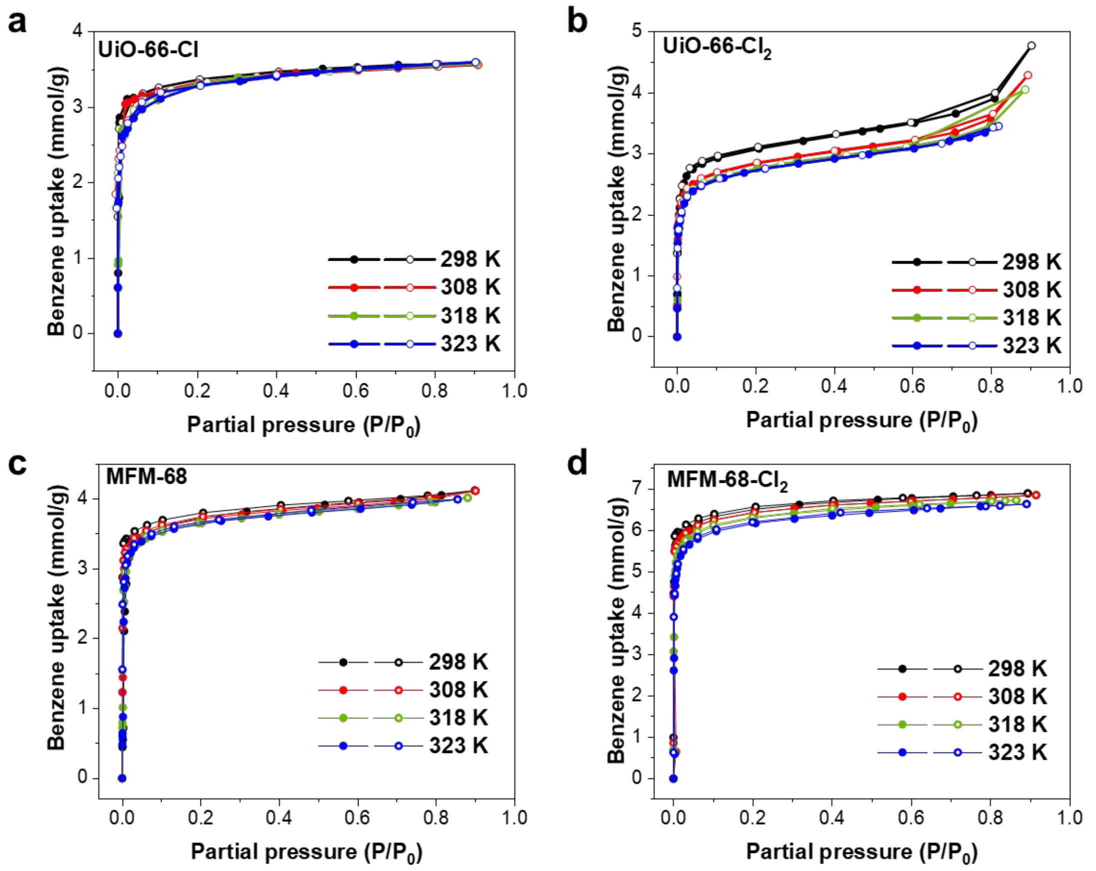

Figure S10. Adsorption isotherms for benzene in (a) UiO-66-Cl, (b) UiO-66-Cl_2, (c) MFM-68 and (d) MFM-68-Cl_2 at 298-323 K (adsorption, solid symbols; desorption, open symbols).

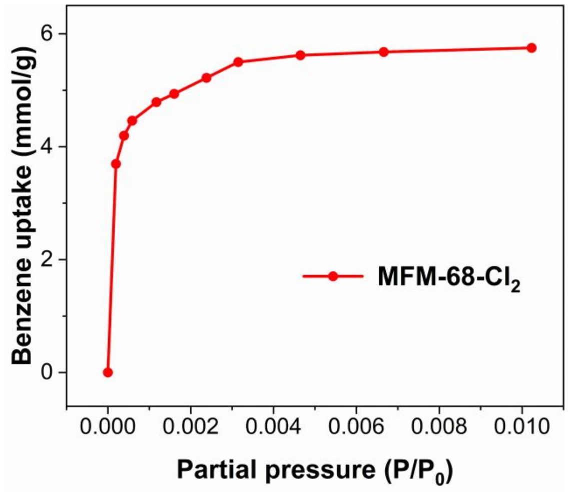

Figure S11. Adsorption isotherms for benzene in MFM-68-Cl_2 at partial pressure of 0-0.01 and 298K.

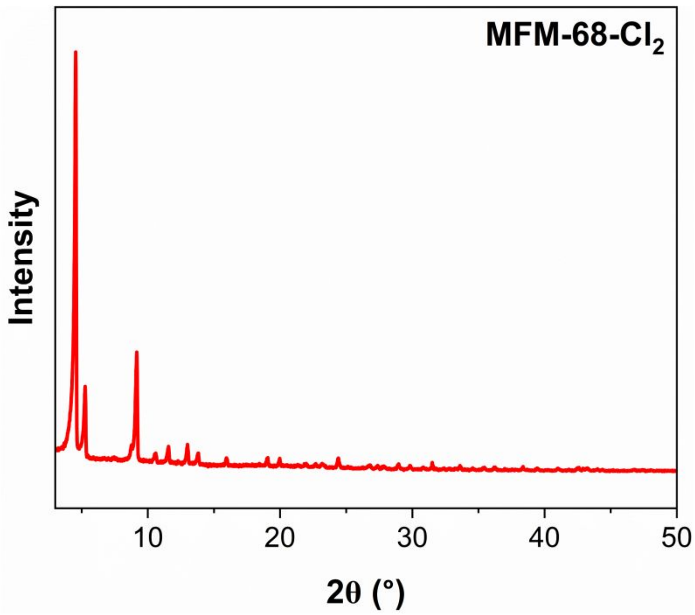

Figure S12. PXRD pattern for MFM-68-Cl_2 after 15 cycles of adsorption/desorption of benzene.

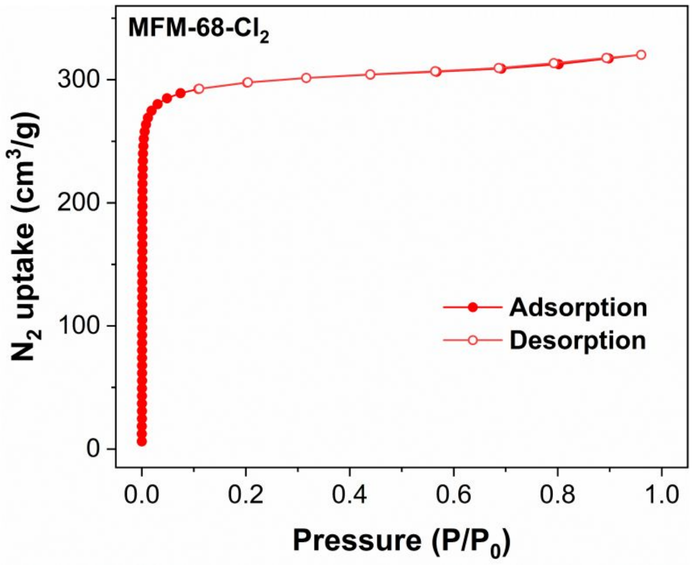

Figure S13. N_2 isotherms at 77 K for MFM-68-Cl_2 after cycling (adsorption, solid symbols; desorption, open symbols).

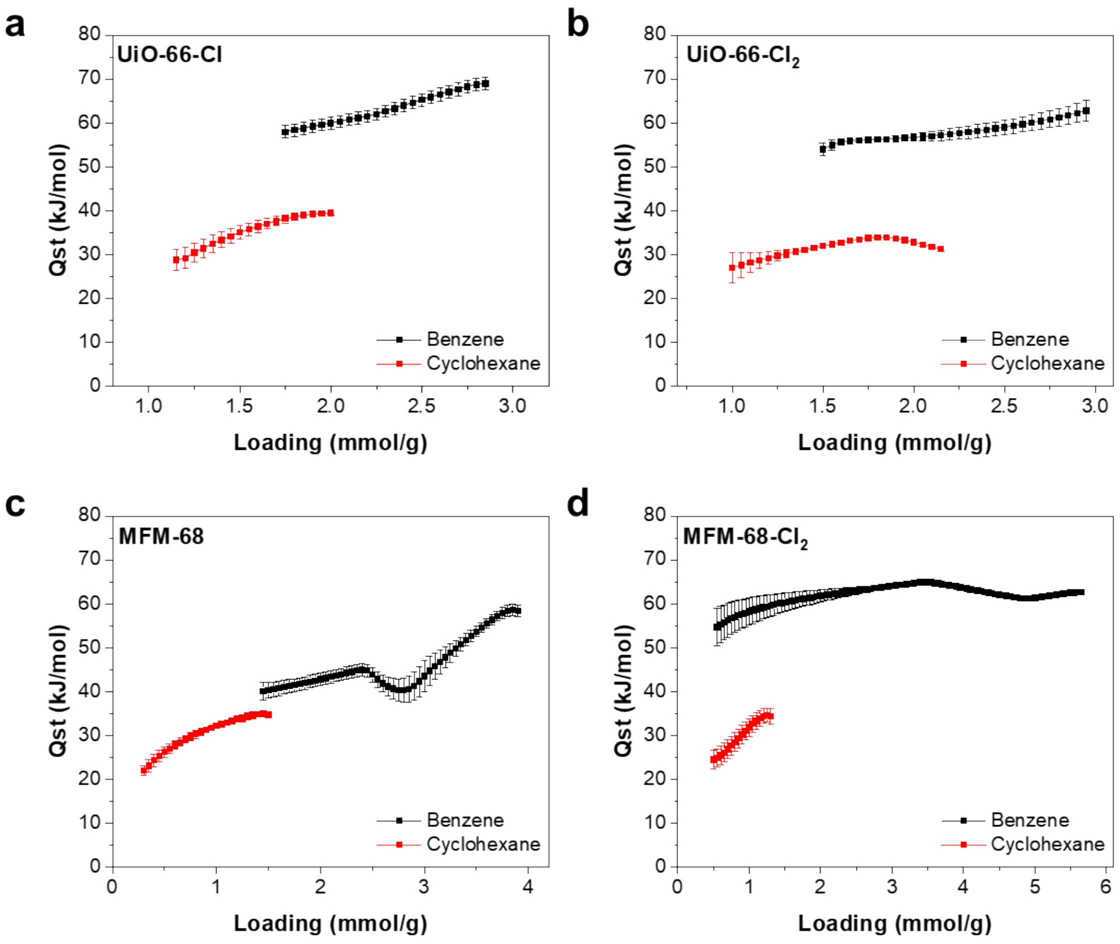

Figure S14. Isosteric heats of adsorption (Q_{st}) of benzene and cyclohexane in (a) UiO-66-Cl, (b) UiO-66-Cl_2, (c) MFM-68 and (d) MFM-68-Cl_2.

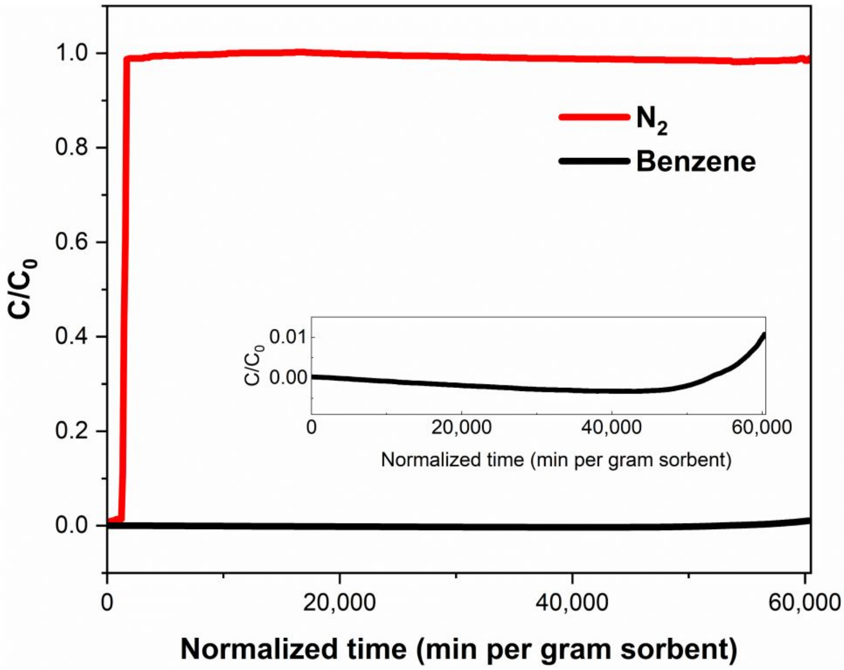

Figure S15. Breakthrough curves for 0.0005% benzene (5 ppm) diluted in air passing through a fixed-bed packed of MFM-68-Cl_2 at 298 K and 1 bar. The inset represents the enlarged benzene breakthrough curve, indicating a 1% breakthrough time of ~60,000 min g^{-1}, corresponding to a dynamic adsorption capacity of ~2.68 mmol g^{-1}.

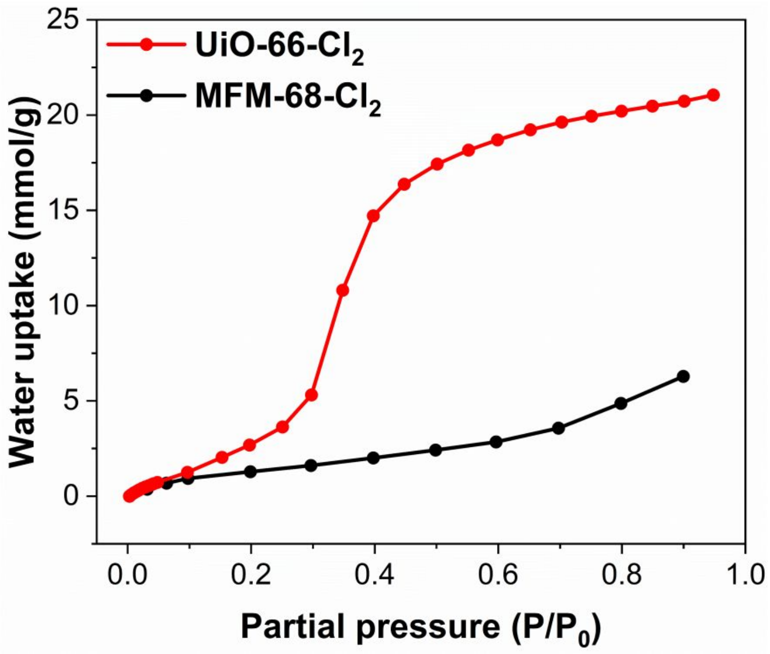

Figure S16. Water adsorption isotherms for UiO-66-Cl_2 and MFM-68-Cl_2 at 298 K.

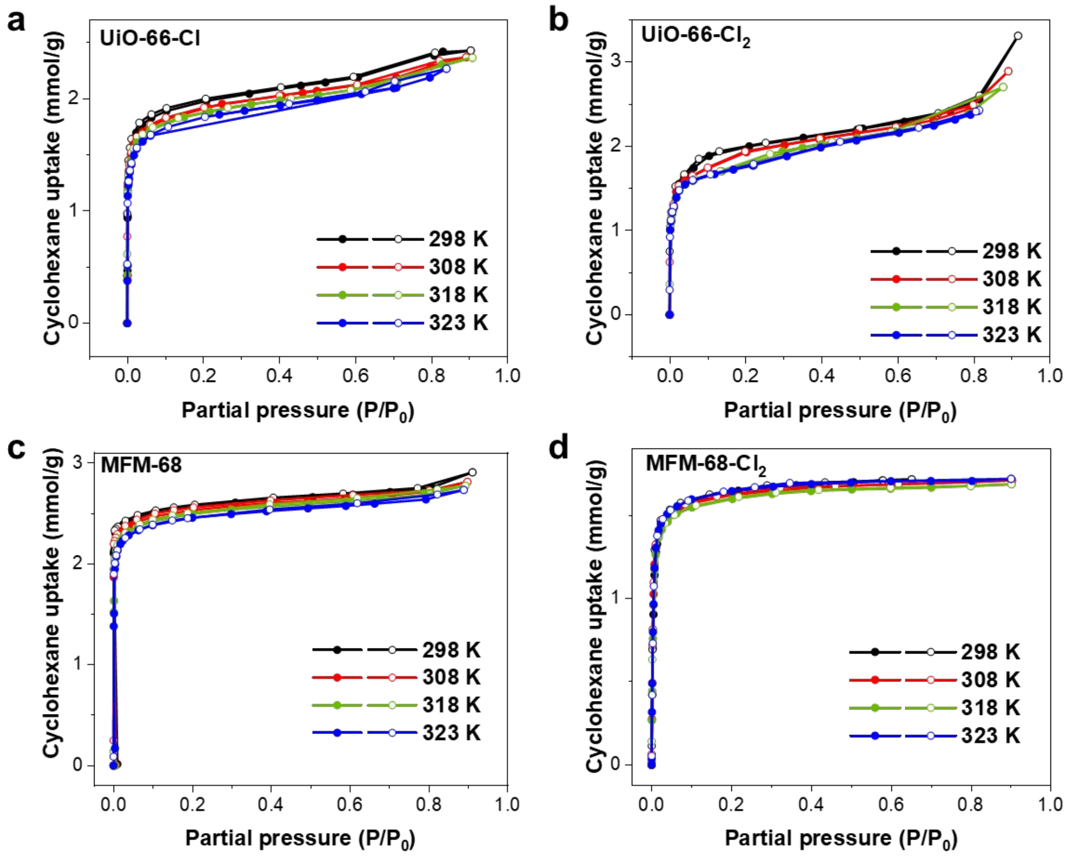

Figure S17. Adsorption isotherms for cyclohexane in (a) UiO-66-Cl, (b) UiO-66-Cl_2, (c) MFM-68 and (d) MFM-68-Cl_2 at 298-323 K (adsorption, solid symbols; desorption, open symbols).

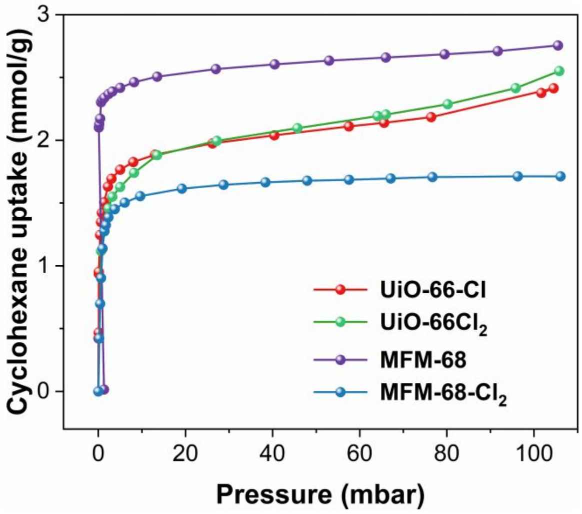

Figure S18. Comparison of adsorption isotherms for cyclohexane in UiO-66-Cl, UiO-66-Cl_2, MFM-68 and MFM-68-Cl_2 at 298 K.

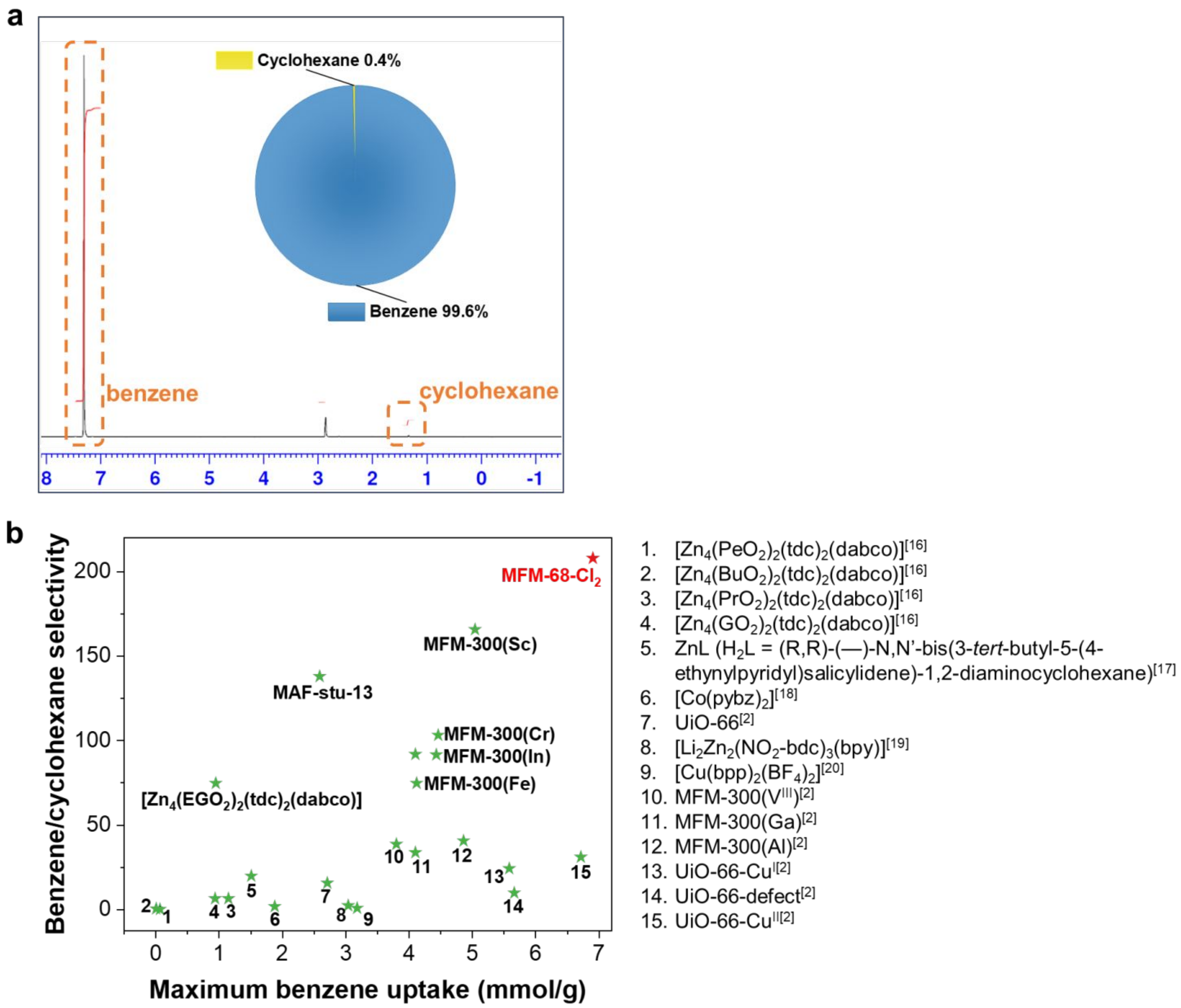

Figure S19. Liquid-phase separation of benzene/cyclohexane/water mixture (v/v/v = 1/1/0.1) in MFM-68-Cl_2. (a) ^1H NMR spectroscopy and relative adsorption of benzene and cyclohexane. (b) Benzene/cyclohexane selectivity of MFM-68-Cl_2 compared with representative sorbents.[2,16-21]

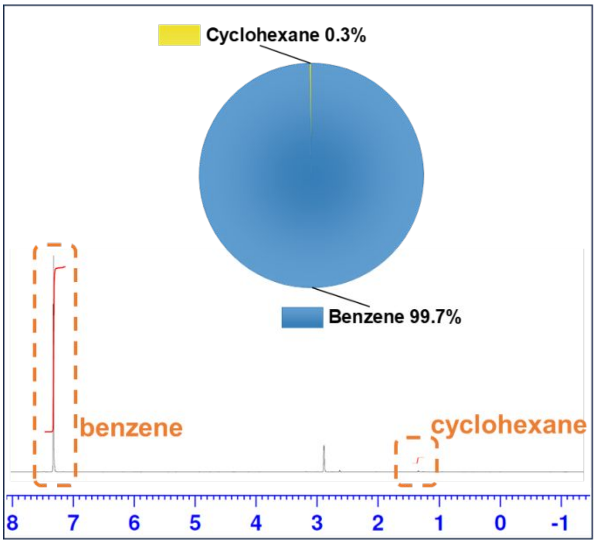

Figure S20. Vapor-phase separation of benzene/cyclohexane/water mixture (v/v/v = 1/1/0.1) in MFM-68-Cl_2. ^{1}H NMR spectroscopy and relative adsorption of benzene and cyclohexane.

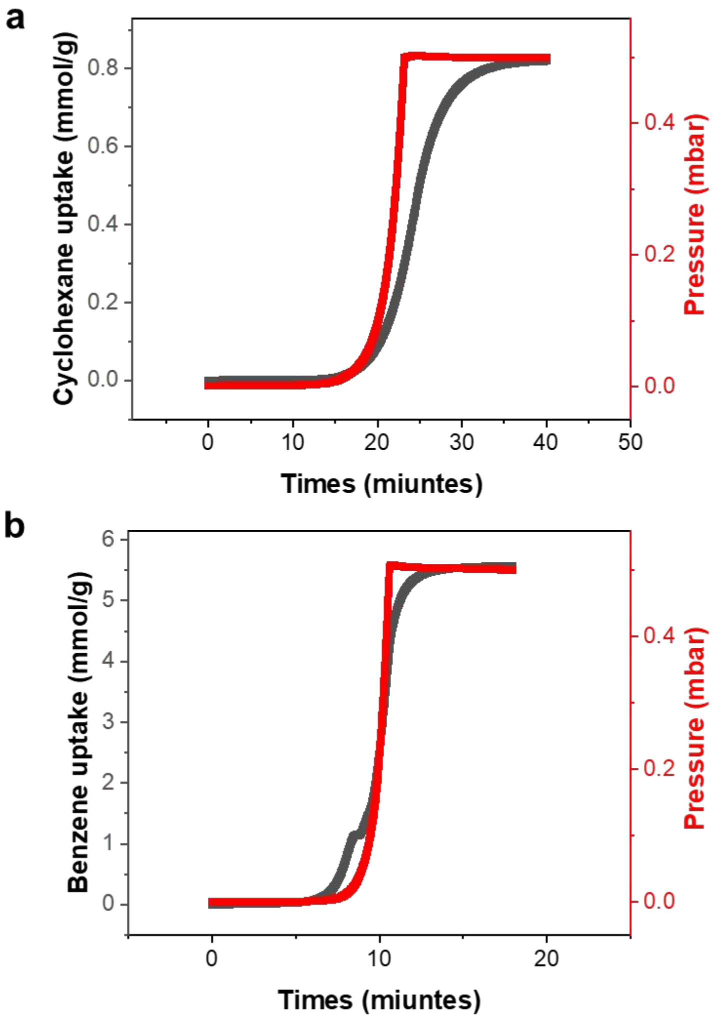

Figure S21. Adsorption kinetics of (a) cyclohexane and (b) benzene in MFM-68-Cl_2 at 298K.

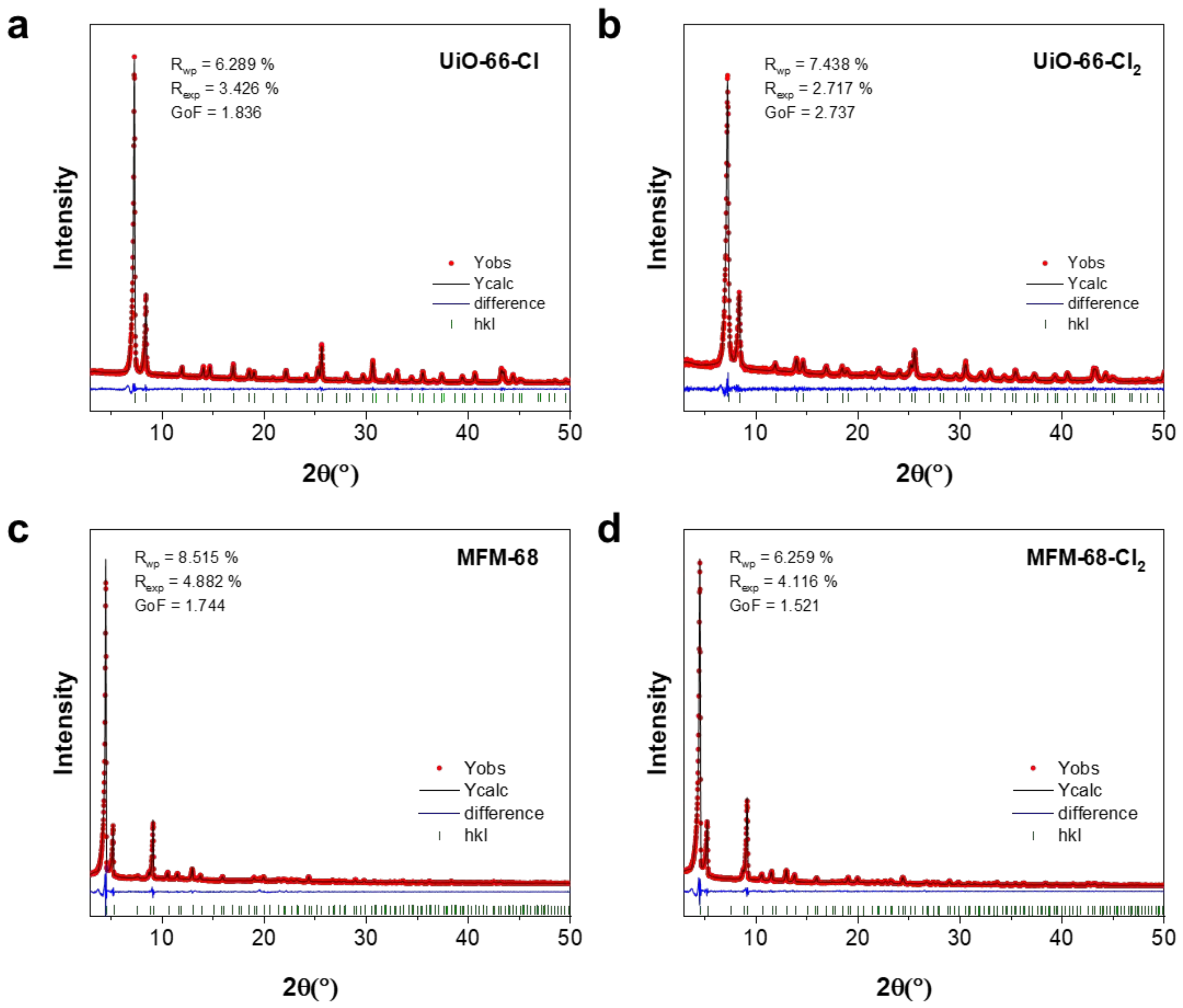

Figure S22. Pawley fitting profiles of PXRD patterns for (a) UiO-66-Cl, (b) UiO-66-Cl_2, (c) MFM-68 and (d) MFM-68-Cl_2 after isotherm measurements.

Table S3. Comparison of state-of-the-art porous materials as sorbents for benzene.

Materials	BET surface area (m^2/g)	Saturated benzene uptake at 298 K (mmol/g)	Benzene uptake at 298 K, 0.12 mbar (mmol/g)	Benzene/cyclohexane selectivity	Ref.
UiO-66-Cl	976	3.59	0.88	1.5 (Benzene/cyclohexane uptake ratio at $P/P_0 \sim 0.9$)	This work
UiO-66-Cl_2	908	4.76	1.43	1.6 (Benzene/cyclohexane uptake ratio at $P/P_0 \sim 0.9$)	This work
MFM-68	1428	4.12	0.49	1.5 (Benzene/cyclohexane uptake ratio at $P/P_0 \sim 0.9$)	This work
MFM-68-Cl_2	920	6.88	4.62	208 (Liquid-phase separation), 277 (Vapor-phase separation), 4 (Benzene/cyclohexane uptake ratio at $P/P_0 \sim 0.9$)	This work
ZJU-620(Al)	1347	4.5	0.7	n/a	11
UiO-66-Cu^{II}	1240	6.71	0.67	31 (Liquid-phase separation)	2
UiO-66	996	3.04	0.63	3 (Liquid-phase separation)	2
BUT-67	984	6.14	1.52	n/a	12
BUT-66	1831	3.97	2.49	n/a	12
BUT-54	1847	5.23	3.01	n/a	13
BUT-53	1096	3.29	2.54	n/a	13
BUT-55	1128	3.56	3.41	n/a	13
BUT-56	811	3.51	3.26	n/a	13
BUT-57	873	6.35	3.15	n/a	13
BUT-58	897	3.44	3.16	n/a	13
MCM-41	1139	15.5	0.072	n/a	12
MFM-300(Sc)	1228	5.04	0.8	166 (Liquid-phase separation)	2
MIL-101(Cr)	2925	15.8	0.66	n/a	12
ZIF-8	1510	3.99	0.024	n/a	12
Carboxen 1000	1017	5.04	2.23	n/a	12
ZJU-520(Al)	2235	12.08	0.52	29.86 (IAST selectivity of benzene/cyclohexane at vapor volume of 50/50)	14
STA-26	1071	4.93	1.14	n/a	15
STA-26-Et	710	4.95	2.21	n/a	15
$[Zn_4(EGO_2)_2(tdc)_2(dabco)]$	587	0.95	n/a	77 (Liquid-phase separation)	16
$[Zn_4(PrO_2)_2(tdc)_2(dabco)]$	420	1.15	n/a	7 (Liquid-phase separation)	16
$[Zn_4(BuO_2)_2(tdc)_2(dabco)]$	301	0.01	n/a	0.7 (Liquid-phase separation)	16
$[Zn_4(PeO_2)_2(tdc)_2(dabco)]$	400	0.07	n/a	0.3 (Liquid-phase	16

				separation)	
ZnL (H_2L = (R,R)-(–)-N,N'-bis(3-tertbutyl-5-(4-ethynylpyridyl)salicylidene)-1,2-diaminocyclohexane)	0.32	1.51	n/a	20 (Liquid-phase separation), 5.1 (Benzene/cyclohexane uptake ratio at $P/P_0 \sim 0.9$)	17
[Co(pybz)$_2$] (pybz = 4-(4-pyridyl)benzoate)	770	1.88	n/a	2 (Liquid-phase separation)	18
[$Li_2Zn_2(NO_2$-bdc)$_3$(bpy)]	742	2.71	n/a	16 (Liquid-phase separation)	19
[Cu(bpp)$_2$(BF_4)$_2$] (bpp = 1,3-bis(4-pyridyl)propane)	11	3.18	n/a	1 (Liquid-phase separation)	20
MAF-stu-13	767	2.59	n/a	138 (Liquid-phase separation)	21
CUB-5	2730	7.6	0.45	1.3 (Benzene/cyclohexane uptake ratio at $P/P_0 \sim 0.11$)	22
MUF-77	3600	17.85	< 0.2	n/a	23
CUB-30	2930	13.39	< 0.2	n/a	23
Zn-TCNQ-bpy (TCNQ = 7,7,8,8-tetracyano-*p*-quinodimethane, bpy =4,4′-bipyridyl)	n/a	3.6	n/a	27 (Vapor-phase separation), 4 (Benzene/cyclohexane uptake ratio at $P/P_0 \sim 1$)	24
MAF-2	n/a	2.64	n/a	21 (Benzene/cyclohexane uptake ratio at $P/P_0 \sim 1$)	25
MAF-24 β	444	10.24	n/a	10.08 (Benzene/cyclohexane uptake ratio at $P/P_0 \sim 0.88$)	26
Mn-TCNQ-bpy (TCNQ = 7,7,8,8-tetracyano-*p*-quinodimethane, bpy =4,4′-bipyridyl)	n/a	3.7	n/a	2.2 (Benzene/cyclohexane uptake ratio at $P/P_0 \sim 1$)	27
$Ni_3(OH)(Ina)_3(BDC)_{1.5}$ (I na = isonicotinate, BDC = 1,4-benzenedicarboxylate)	1255	2.9	n/a	145 (Benzene/cyclohexane uptake ratio at $P/P_0 \sim 1$)	28
Mn-MOF-74	1500	9.38	n/a	37.5 (Benzene/cyclohexane uptake ratio at P = 13.5 kPa)	29
MFOF-1	2287	5.23	n/a	1.5 (Benzene/cyclohexane uptake ratio at $P/P_0 \sim 1$)	30
Cd-dtztp (H_4dtztp = 2,5-di(2H-tetrazol-5-yl)terephthalic acid)	576	6.52	n/a	6 (Benzene/cyclohexane uptake ratio at $P/P_0 \sim 1$)	31

6. Rietveld Refinement of SXPD Patterns

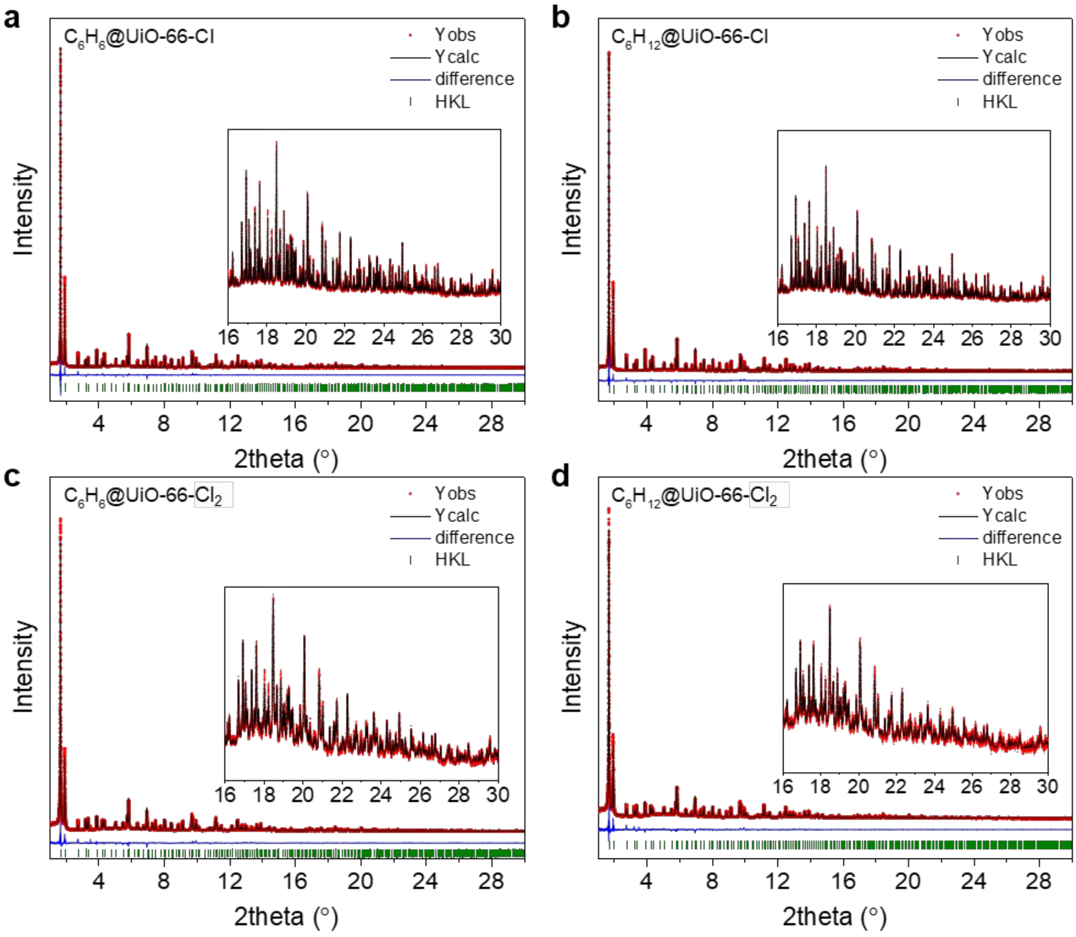

Figure S23. Rietveld refinement of SXPD patterns (λ = 0.354243 Å, ESRF, ID22) of (a) C_6H_6@UiO-66-Cl, (b) C_6H_{12}@ UiO-66-Cl, (c) C_6H_6@UiO-66-Cl_2 and (d) C_6H_{12}@UiO-66-Cl_2. Insets are enlarged fitting range of 16-30°.

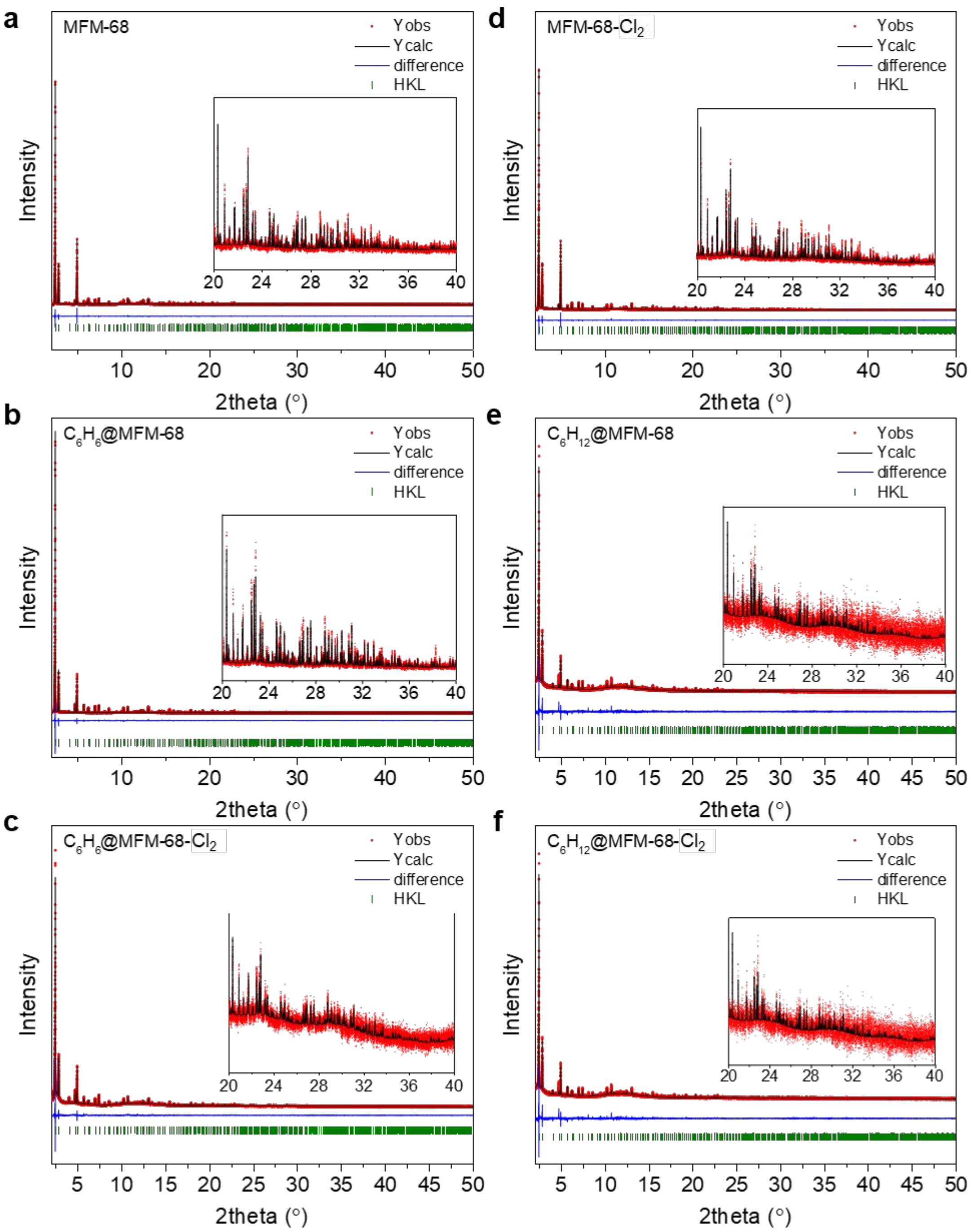

Figure S24. Rietveld refinement of SXPD patterns (λ = 0.825894 Å, Diamond Light Source, I11) of (a) bare MFM-68, (b) C_6H_6@MFM-68, (c) C_6H_6@MFM-68-Cl, (d) bare MFM-68-Cl_2, (e) C_6H_{12}@MFM-68, (f) C_6H_{12}@MFM-68-Cl_2. Insets are enlarged fitting range of 20-40°.

7. Crystallographic Data

Table S4. Crystallographic data from Rietveld refinement.

**Samples**	**C_6H_6@UiO-66-Cl**	**C_6H_6@UiO-66-Cl_2**	**C_6H_{12}@UiO-66-Cl**	**C_6H_{12}@UiO-66-Cl_2**
CCDC No.	2329520	2329521	2329522	2329524
Formula	$[Zr_6O_4(OH)_4(BDC–Cl)_6]$ $(C_6H_6)_{6.048}$	$[Zr_6O_4(OH)_4(BDC–Cl_2)_6]$ $(C_6H_6)_{6.13}$	$[Zr_6O_4(OH)_4(BDC–Cl)_6]$ $(C_6H_{12})_{5.822}$	$[Zr_6O_4(OH)_4(BDC–Cl_2)_6]$ $(C_6H_{12})_{5.395}$
Crystal system	Cubic	Cubic	Cubic	Cubic
Space group	*F m-3m* (225)	*F m-3m* (225)	*F m-3m* (225)	*F m-3m* (225)
$a = b = c$ /(Å)	20.79252(2)	20.82469(4)	20.79015(2)	20.82041(4)
Vol. (Å^3)	8989.21(2)	9031.00(5)	8986.13(2)	9025.43(6)
ρ (calc) g/cm^3	1.661	1.880	1.745	1.863
Diffractometer	ESRF, ID22	ESRF, ID22	ESRF, ID22	ESRF, ID22
Radiation	Synchrotron	Synchrotron	Synchrotron	Synchrotron
Method	High-resolution	High-resolution	High-resolution	High-resolution
Temperature	298 K	298 K	298 K	298 K
Refinement range	1–30° (λ=0.35424 Å)	1–30° (λ=0.35424 Å)	1–30° (λ=0.35424 Å)	1–30° (λ=0.35424 Å)
$R_{wp}/R_{exp}/R_p$ (%)	4.695/2.100/4.054	4.642/ 2.015/ 4.058	4.665/ 1.927/ 4.258	4.413/ 2.627/ 3.971
GoF (χ^2)	2.235	2.303	2.421	1.680
**Samples**	**MFM-68**	**MFM-68-Cl_2**	**C_6H_6@MFM-68**	**C_6H_{12}@MFM-68**
CCDC No.	2329525	2329523	2329518	2329519
Formula	$[Zr_6O_4(OH)_4(Teran)_6]$ $(H_2O)_{50.98}$	$[Zr_6O_4(OH)_4(Teran-Cl_2)_6]$ $(H_2O)_{38.16}$	$[Zr_6O_4(OH)_4(Teran)_6]$ $(H_2O)_{6.288}$ $(C_6H_6)_{9.648}$	$[Zr_6O_4(OH)_4(Teran)_6]$ $(C_6H_{12})_{11.986}$
Crystal system	Cubic	Cubic	Cubic	Cubic
Space group	*F m-3m* (225)	*F m-3m* (225)	*F m-3m* (225)	*F m-3m* (225)
$a = b = c$ /(Å)	33.0734(2)	33.0730(2)	33.07015(14)	33.01100(7)
Vol. (Å^3)	36177.2(8)	36176.0(7)	36166.7(5)	35972.9(2)
ρ (calc) g/cm^3	0.954	0.955	0.980	0.995
Diffractometer	DLS, I11	DLS, I11	DLS, I11	DLS, I11
Radiation	Synchrotron	Synchrotron	Synchrotron	Synchrotron
Method	High-resolution	High-resolution	High-resolution	High-resolution
Temperature	298 K	298 K	298 K	298 K
Refinement range	2–50° (λ=0.82683 Å)	2–50° (λ=0.82683 Å)	2–50° (λ=0.82683 Å)	2–50° (λ=0.82683 Å)
$R_{wp}/R_{exp}/R_p$ (%)	7.314/5.368/5.415	7.084/4.850/5.180	8.521/3.543/6.238	10.313/4.260/8.096
GoF (χ^2)	1.362	1.460	2.404	2.421
**Samples**	**C_6H_6@MFM-68-Cl_2**	**C_6H_{12}@MFM-68-Cl_2**		
CCDC No.	2329468	2205380		
Formula	$[Zr_6O_4(OH)_4(Teran-Cl_2)_6]$ $(C_6H_6)_{17.52}$	$[Zr_6O_4(OH)_4(Teran-Cl_2)_6]$ $(C_6H_{12})_{10.56}$		
Crystal system	Cubic	Cubic		
Space group	*F m-3m* (225)	*F m-3m* (225)		
$a = b = c$ /(Å)	33.00241(12)	33.03403(15)		
Vol. (Å^3)	35944.9(4)	36048.3(5)		
ρ (calc) g/cm^3	1.139	1.011		

Diffractometer	DLS, I11	DLS, I11
Radiation	Synchrotron	Synchrotron
Method	High-resolution	High-resolution
Temperature	298 K	298 K
Refinement range	2–50° (λ=0.82683 Å)	2–50° (λ=0.82683 Å)
$R_{wp}/R_{exp}/R_p$ (%)	7.334/3.968/5.724	10.135/5.478/7.720
GoF (χ^2)	1.848	1.850

8. Views of Crystal Structures

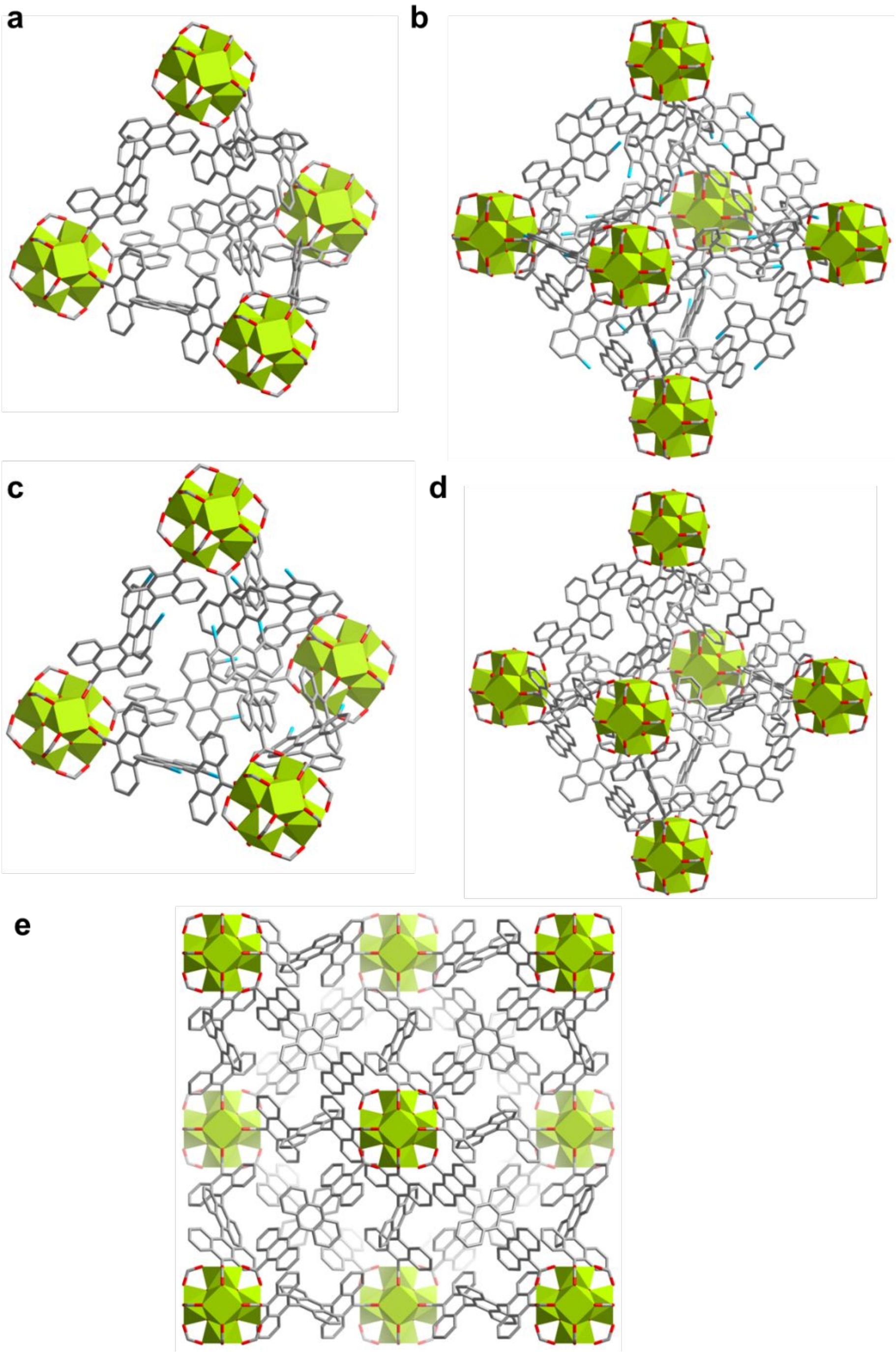

Figure S25. Views of crystal structures of MFM-68 and MFM-68-Cl_2. (a) Tetrahedral and (b) octahedral cages in MFM-68; (c) tetrahedral and (d) octahedral cages in MFM-68-Cl_2; (e) view of unit cell along *a*-axis. Hydrogen omitted for clarity.

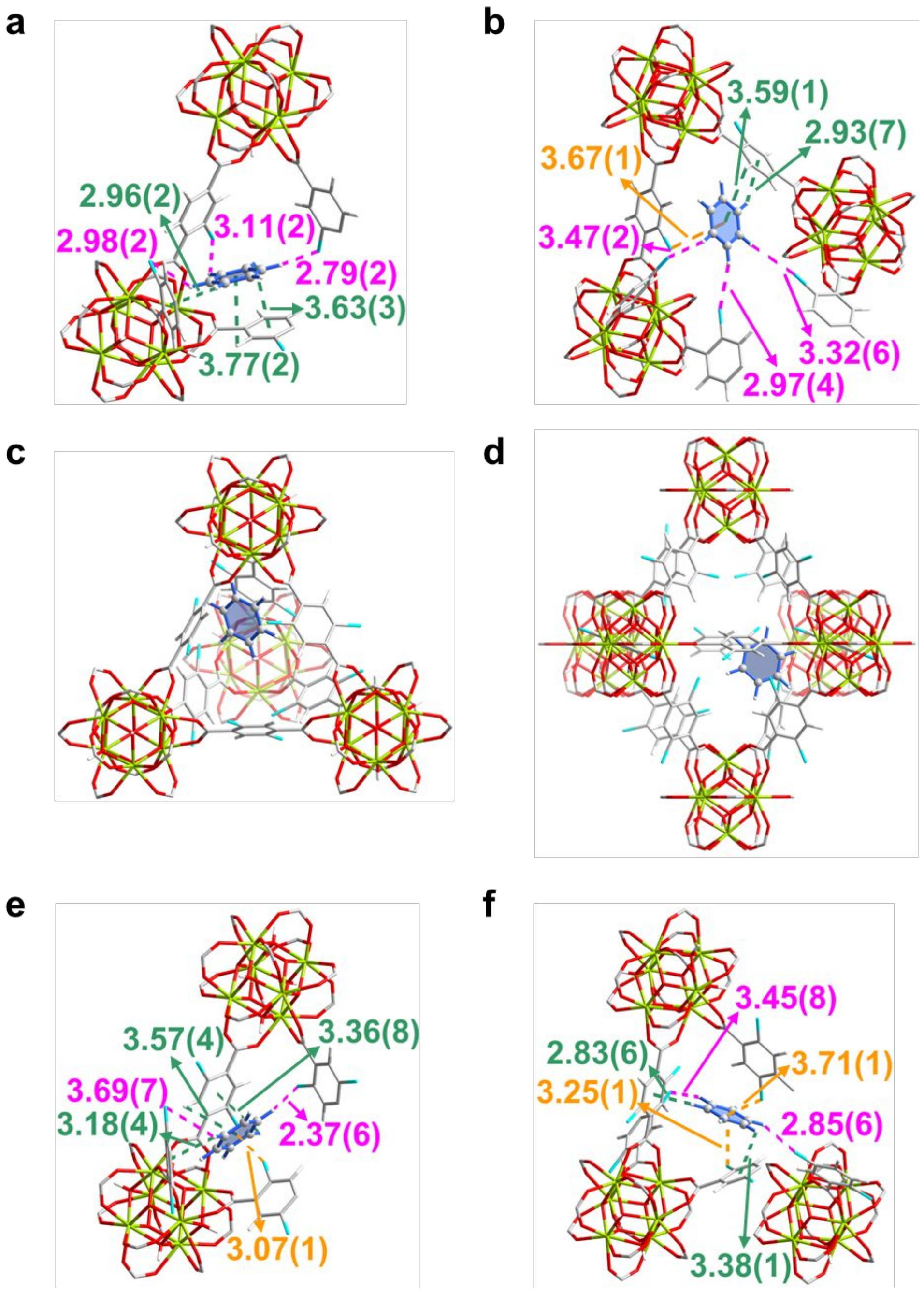

Figure S26. Views of the structural models for adsorbed benzene in UiO-66-Cl and UiO-66-Cl_2. Detailed views of (a) site Ⅰ and (b) site Ⅱ for benzene in UiO-66-Cl. Views of binding sites for benzene in (c) tetrahedral cage and (d) octahedral cage of UiO-66-Cl_2. Detailed views of (e) binding site Ⅰ and (f) binding site Ⅱ for benzene in UiO-66-Cl_2. Colour code: Zr, lime; C, gray; O, red; H, white; Cl, blue.

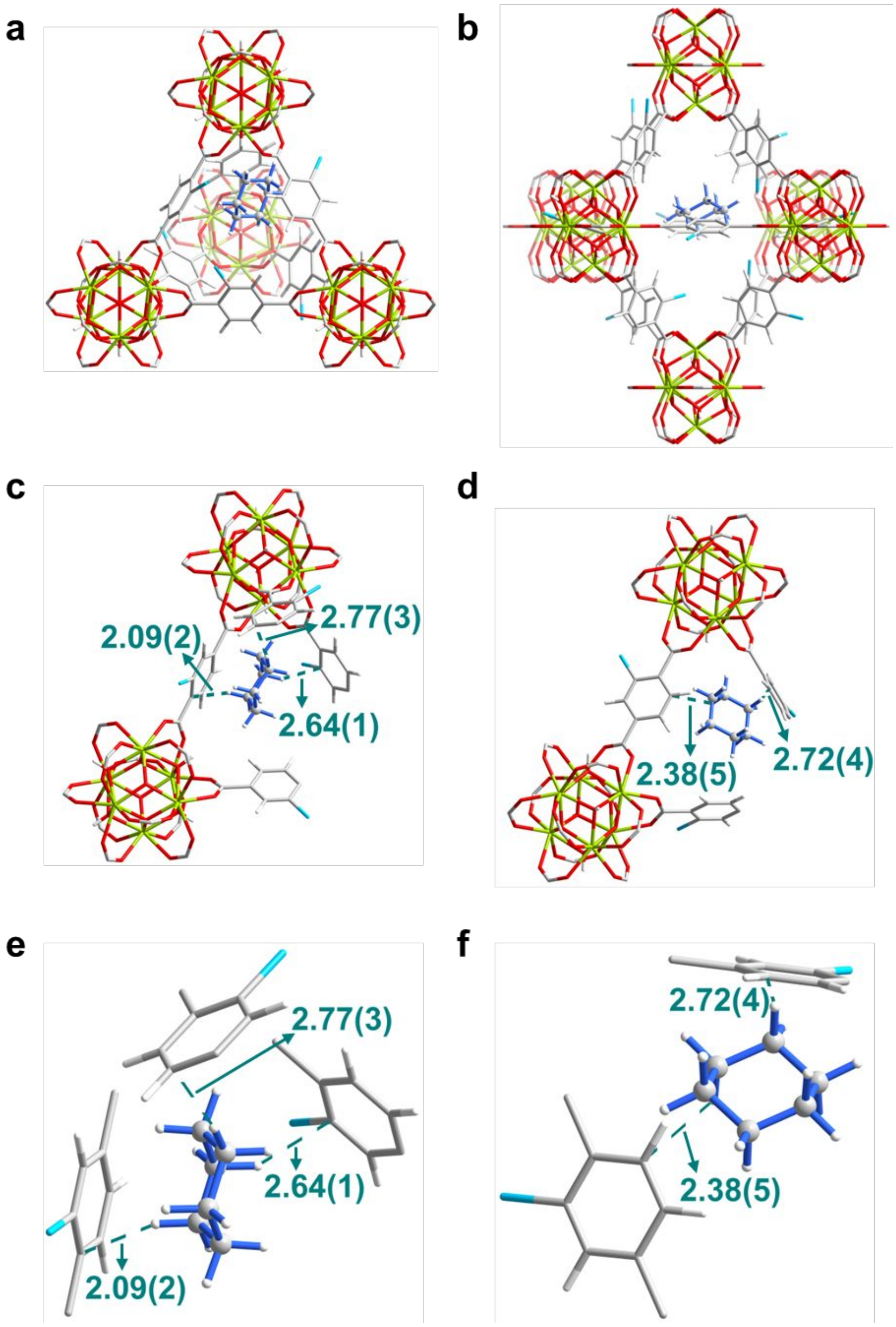

Figure S27. Views of the structural models for adsorbed cyclohexane in UiO-66-Cl. Views of binding site positions for cyclohexane in (a) tetrahedral cage and (b) octahedral cage of UiO-66-Cl. Detailed views of (c and e) binding site Ⅰ’, and (d and f) binding site Ⅱ’ for cyclohexane in UiO-66-Cl. Colour code: Zr, lime; C, gray; O, red; H, white; Cl, blue.

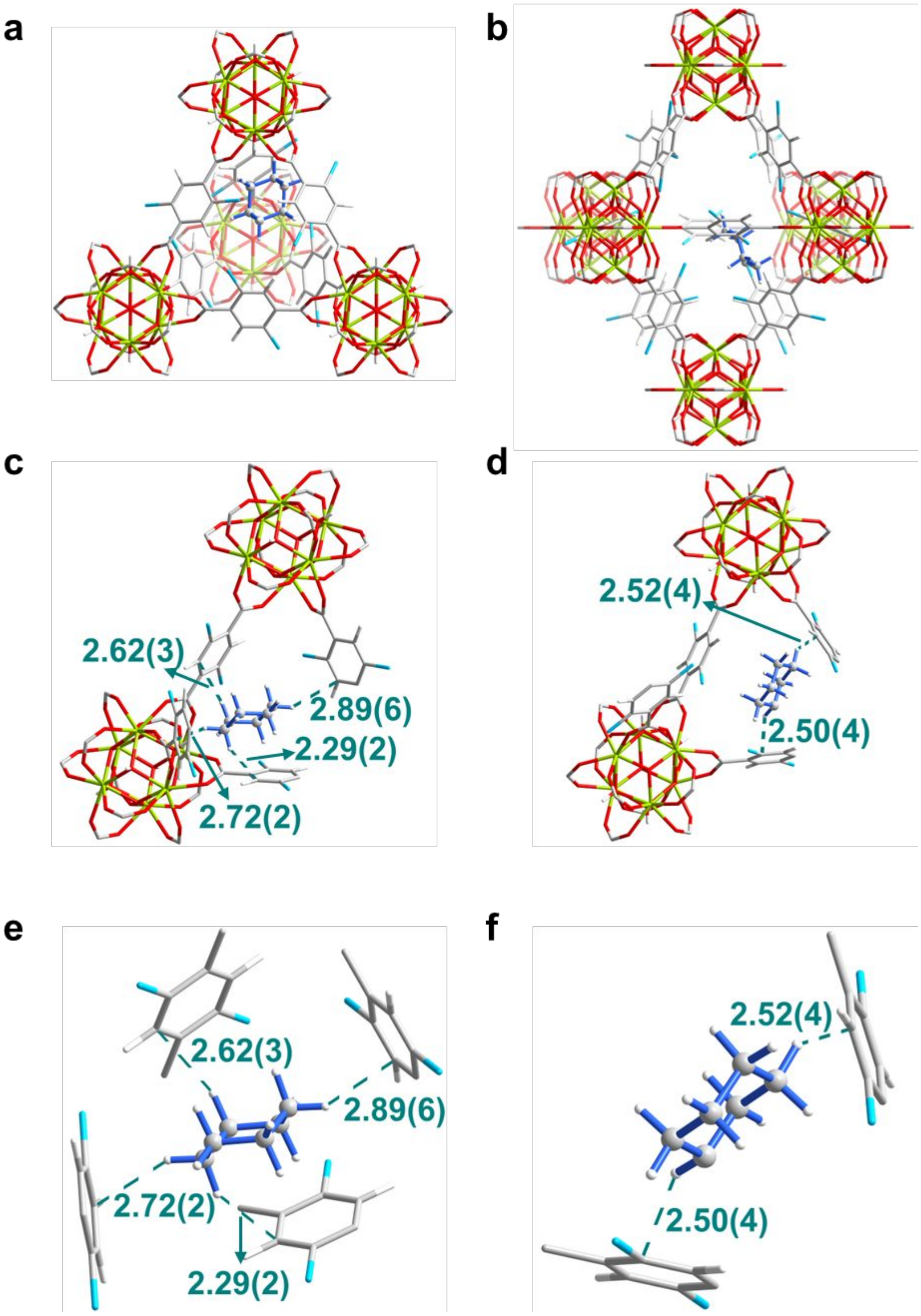

Figure S28. Views of the structural models for adsorbed cyclohexane in UiO-66-Cl_2. Views of binding site positions for cyclohexane in (a) tetrahedral cage and (b) octahedral cage of UiO-66-Cl_2. Detailed views of (c and e) binding site Ⅰ’, and (d and f) binding site Ⅱ’ for cyclohexane in UiO-66-Cl_2. Colour code: Zr, lime; C, gray; O, red; H, white; Cl, blue.

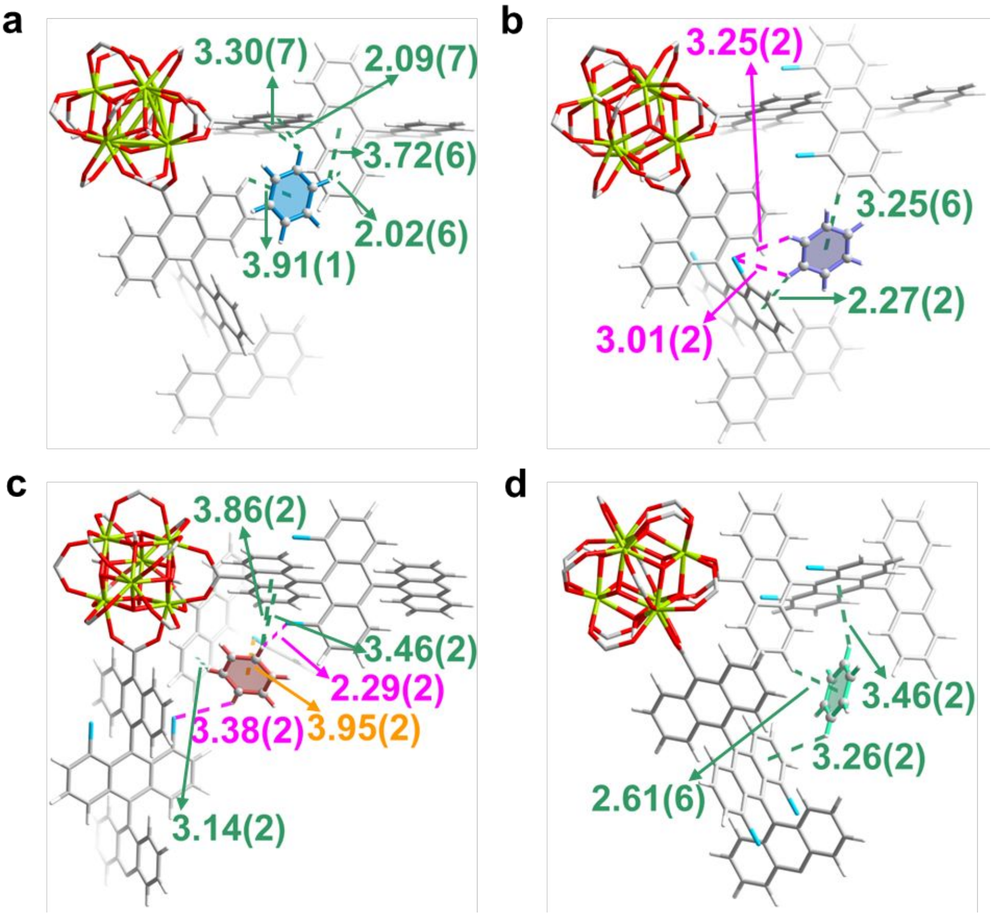

Figure S29. Views of the structural models for adsorbed benzene in MFM-68 and MFM-68-Cl_2. (a) Detailed views of the single binding site for benzene in MFM-68. Detailed views of (b) binding site Ⅰ, (c) site Ⅱ and (d) site Ⅲ for benzene in MFM-68-Cl_2. Colour code: Zr, lime; C, gray; O, red; H, white; Cl, blue.

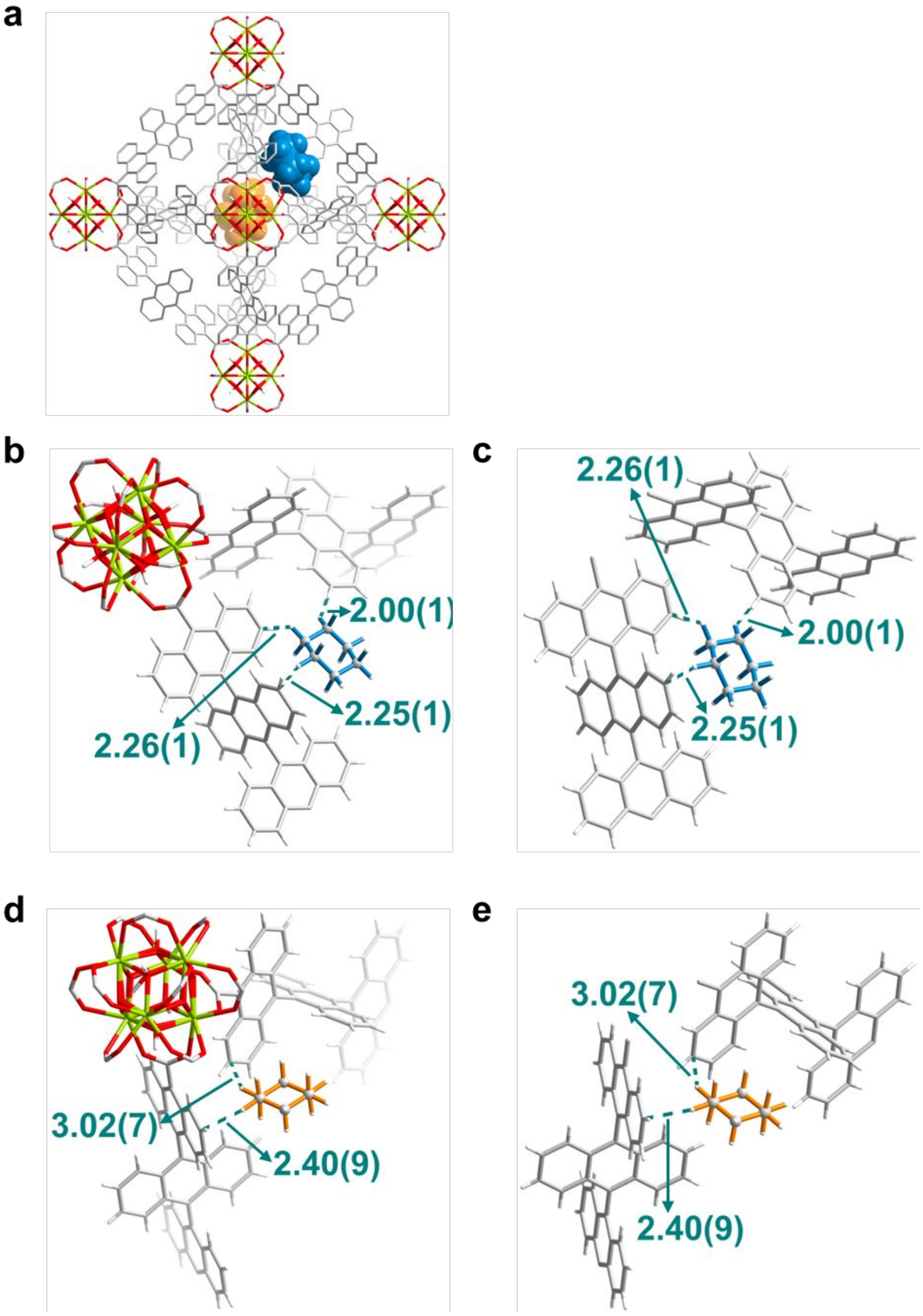

Figure S30. Views of the structural models for adsorbed cyclohexane in MFM-68. (a) Views of binding site positions for cyclohexane in the octahedral cage of MFM-68. Detailed views of (b and c) binding site Ⅰ’, and (d and e) site Ⅱ’ for benzene in MFM-68. Colour code: Zr, lime; C, gray; O, red; H, white.

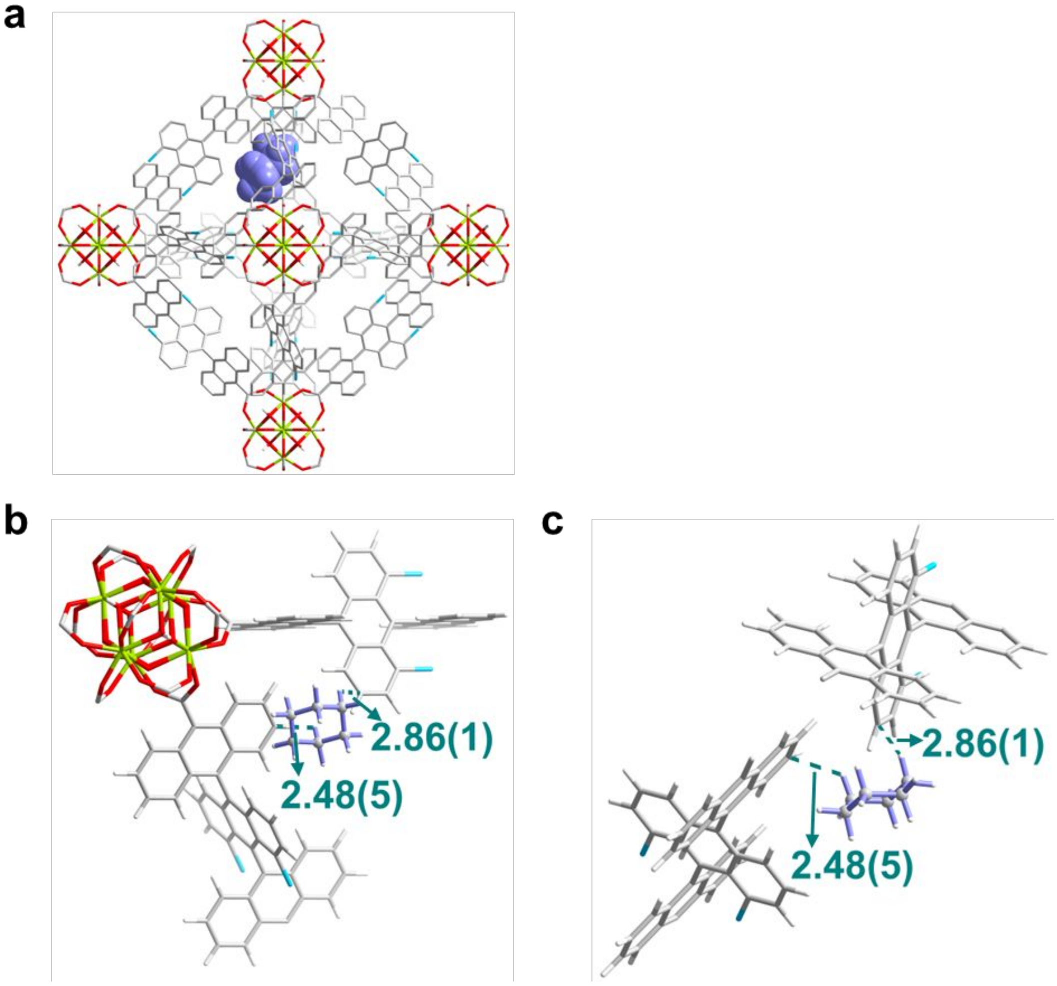

Figure S31. Views of the structural models for adsorbed cyclohexane in MFM-68-Cl_2. (a) Views of binding site positions for cyclohexane in the octahedral cage of MFM-68-Cl_2. (b and c) Detailed views of the single binding site for benzene in MFM-68-Cl_2. Colour code: Zr, lime; C, gray; O, red; H, white; Cl, blue.

9. *In Situ* FTIR Spectra

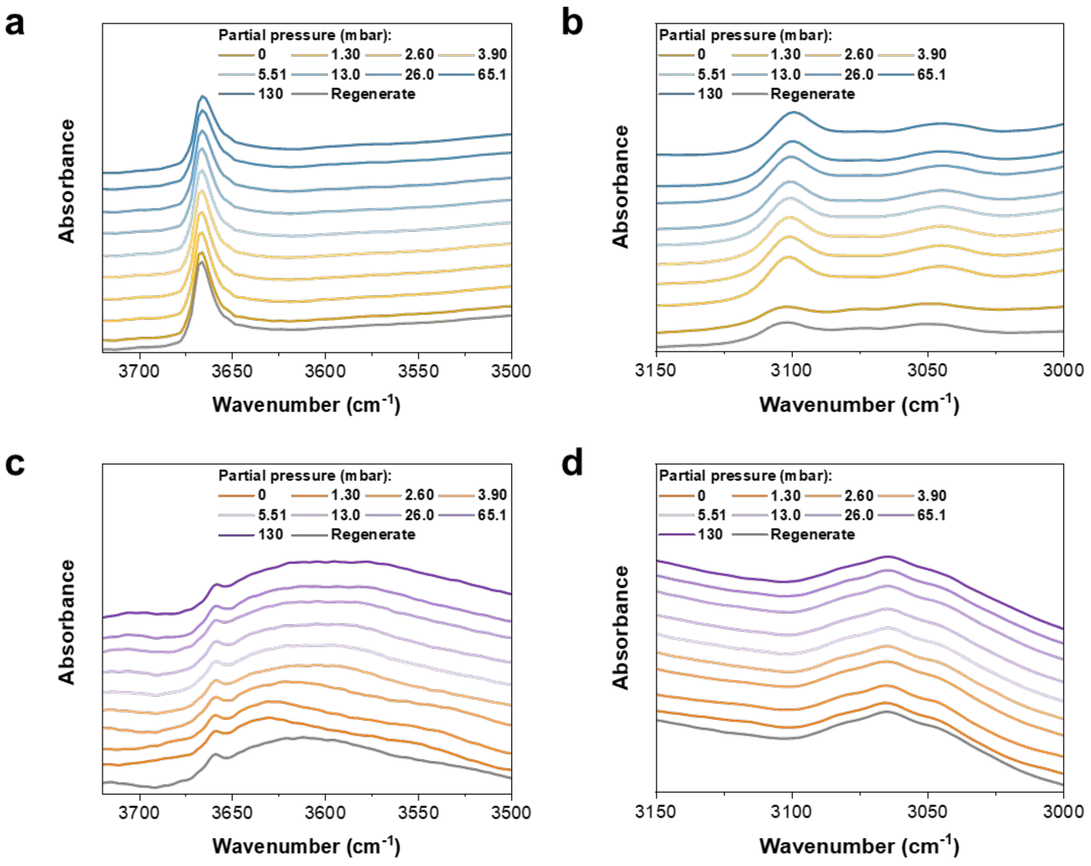

Figure S32. *In situ* FTIR spectra of UiO-66-Cl_2 and MFM-68-Cl_2. (a and c) The v(OH) and (b and d) CH stretching region of (a and b) UiO-66-Cl_2 and (c and d) MFM-68-Cl_2 at partial pressures of cyclohexane from 0–130 mbar (diluted in dry N_2) at 298 K and after regeneration at 353 K with dry N_2 flow.

10. INS Spectra

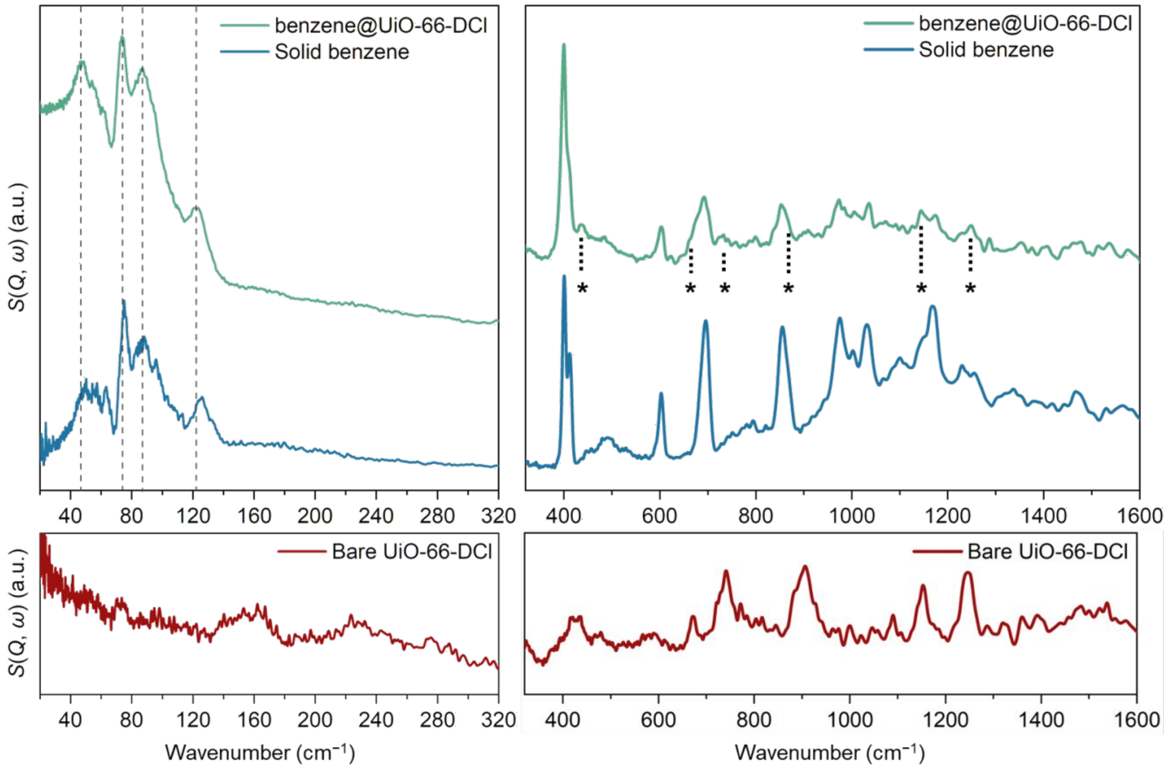

Figure S33. INS spectra of bare (red) and benzene-loaded (green) UiO-66-Cl_2 compared to solid benzene (blue). The spectrum is divided into lattice mode region (20–320 cm^{-1}, left) and vibration mode region (320–1600 cm^{-1}, right) for clarity. The peaks originated from the framework in the vibration mode region are highlighted with an asterisk.

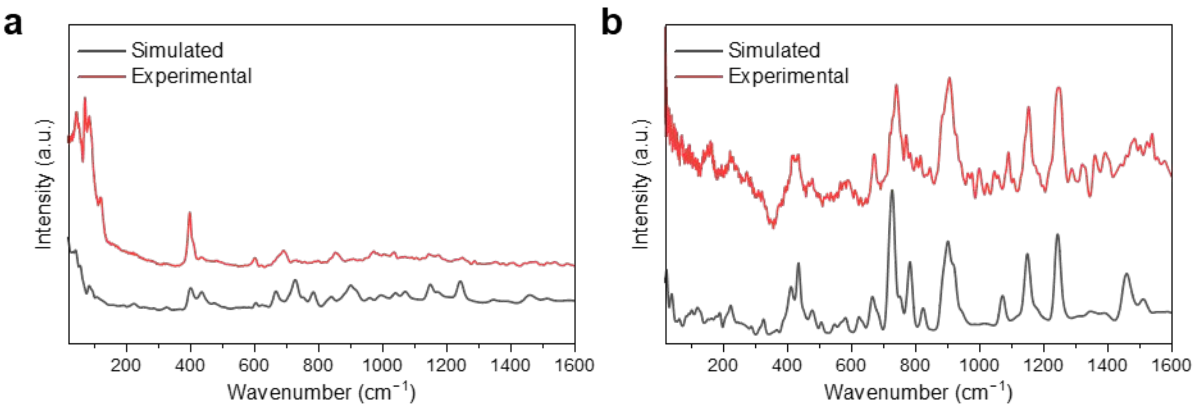

Figure S34. Comparison of DFT-simulated INS spectra and experimental INS spectra of (a) bare UiO-66-Cl_2 and (b) benzene-loaded UiO-66-Cl_2.

11. References

[1] Biswas, S.; Van Der Voort, P. A General Strategy for the Synthesis of Functionalised UiO - 66 Frameworks: Characterisation, Stability and CO_2 Adsorption Properties. *Eur. J. Inorg. Chem.* **2013**, *2013*, 2154–2160.

[2] Han, Y.; Chen, Y.; Ma, Y.; Bailey, J.; Wang, Z.; Lee, D.; Sheveleva, A. M.; Tuna, F.; McInnes, E. J. L.; Frogley, M. D.; Day, S. J.; Thompson, S. P.; Spencer, B. F.; Nikiel, M.; Manuel, P.; Crawshaw, D.; Schröder, M.; Yang, S. Control of the Pore Chemistry in Metal-Organic Frameworks for Efficient Adsorption of Benzene and Separation of Benzene/Cyclohexane. *Chem* **2023**, 9, 739–754.

[3] Yao, L.-Y.; Yam, V. W.-W. Dual Emissive Gold(I)–Sulfido Cluster Framework Capable of Benzene–Cyclohexane Separation in the Solid State Accompanied by Luminescence Color Changes. *J. Am. Chem. Soc.* **2021**, *143*, 2558–2566.

[4] Kresse, G.; Furthmüller, J. Efficient Iterative Schemes forAb InitioTotal-Energy Calculations Using a Plane-Wave Basis Set. Phys. Rev. B Condens. *Matter* **1996**, *54*, 11169–11186.

[5] Blöchl, P. E. Projector Augmented-Wave Method. Phys. Rev. B Condens. *Matter* **1994**, *50* , 17953–17979.

[6] Kresse, G.; Joubert, D. From Ultrasoft Pseudopotentials to the Projector Augmented-Wave Method. Phys. Rev. B Condens. *Matter* **1999**, *59*, 1758–1775.

[7] Perdew, J. P.; Burke, K.; Ernzerhof, M. Generalized Gradient Approximation Made Simple. *Phys. Rev. Lett.* **1996**, *77*, 3865–3868.

[8] Klimeš, J.; Bowler, D. R.; Michaelides, A. Chemical Accuracy for the van Der Waals Density Functional. *J. Phys. Condens. Matter* **2010**, *22*, 022201.

[9] Togo, A.; Tanaka, I. First Principles Phonon Calculations in Materials Science. *Scr. Mater.* **2015**, *108*, 1–5.

[10] Cheng, Y. Q.; Daemen, L. L.; Kolesnikov, A. I.; Ramirez-Cuesta, A. J. Simulation of Inelastic Neutron Scattering Spectra Using OCLIMAX. *J. Chem. Theory Comput.* **2019**, *15*, 1974–1982.

[11] Hu, L.; Wu, W.; Gong, L.; Zhu, H.; Jiang, L.; Hu, M.; Lin, D.; Yang, K. A Novel Aluminum-based Metal-organic Framework with Uniform Micropores for Trace BTEX Adsorption. *Angew. Chem. Int. Ed Engl.* **2023**, *62*, e202215296.

[12] Xie, L.-H.; Liu, X.-M.; He, T.; Li, J.-R. Metal-Organic Frameworks for the Capture of Trace Aromatic Volatile Organic Compounds. *Chem* **2018**, *4*, 1911–1927.

[13] He, T.; Kong, X.-J.; Bian, Z.-X.; Zhang, Y.-Z.; Si, G.-R.; Xie, L.-H.; Wu, X.-Q.; Huang, H.; Chang, Z.; Bu, X.-H.; Zaworotko, M. J.; Nie, Z.-R.; Li, J.-R. Trace Removal of Benzene Vapour Using Double-Walled Metal–Dipyrazolate Frameworks. *Nat. Mater.* **2022**, *21*, 689–695.

[14] Hu, L.; Wu, W.; Hu, M.; Jiang, L.; Lin, D.; Wu, J.; Yang, K. Double-Walled Al-Based MOF with Large Microporous Specific Surface Area for Trace Benzene Adsorption. *Nat. Commun.* **2024**, *15*, 3204.

[15] Lv, J.-A.; Tang, Z.-L.; Liu, Y.-H.; Zhao, R.-C.; Xie, L.-H.; Liu, X.-M.; Li, J.-R. Interior and Exterior Surface Modification of Zr-Based Metal–Organic Frameworks for Trace Benzene Removal. *Inorg. Chem.* **2024**, *63*, 4249–4259.

[16] Lysova, A. A.; Samsonenko, D. G.; Dorovatovskii, P. V.; Lazarenko, V. A.; Khrustalev, V. N.; Kovalenko, K. A.; Dybtsev, D. N.; Fedin, V. P. Tuning the Molecular and Cationic Affinity in a Series of Multifunctional Metal–Organic Frameworks Based on Dodecanuclear Zn(II) Carboxylate Wheels. *J. Am. Chem. Soc.* **2019**, *141*, 17260–17269.

[17] Li, G.; Zhu, C.; Xi, X.; Cui, Y. Selective Binding and Removal of Organic Molecules in a Flexible Polymeric Material with Stretchable Metallosalen Chains. *Chem. Commun. (Camb.)* **2009**, *16*, 2118.

[18] Sapianik, A. A.; Kovalenko, K. A.; Samsonenko, D. G.; Barsukova, M. O.; Dybtsev, D. N.; Fedin, V. P. Exceptionally Effective Benzene/Cyclohexane Separation Using a Nitro-Decorated Metal–Organic Framework. *Chem. Commun. (Camb.)* **2020**, *56*, 8241–8244.

[19] Ye, C.-R.; Wang, W.-J.; Chen, W.; Xiao, Y.; Zhang, H.-F.; Dai, B.-L.; Chen, S.-H.; Wu, X.-D.; Li, M.; Huang, X.-C. Harnessing Shape Complementarity for Upgraded Cyclohexane Purification through Adaptive Bottlenecked Pores in an Imidazole‐containing MOF. *Angew. Chem. Int. Ed Engl.* **2021**, *60*, 23590–23595.

[20] Kondo, A.; Suzuki, T.; Kotani, R.; Maeda, K. Liquid/Vapor-Induced Reversible Dynamic Structural Transformation of a Three-Dimensional Cu-Based MOF to a One-Dimensional MOF Showing Gate Adsorption. *Dalton Trans.* **2017**, *46*, 6762–6768.

[21] Zeng, M.-H.; Tan, Y.-X.; He, Y.-P.; Yin, Z.; Chen, Q.; Kurmoo, M. A Porous 4-Fold-Interpenetrated Chiral Framework Exhibiting Vapochromism, Single-Crystal-to-Single-Crystal Solvent Exchange, Gas Sorption, and a Poisoning Effect. *Inorg. Chem.* **2013**, *52*, 2353–2360.

[22] Macreadie, L. K.; Mensforth, E. J.; Babarao, R.; Konstas, K.; Telfer, S. G.; Doherty, C. M.; Tsanaktsidis, J.; Batten, S. R.; Hill, M. R. CUB-5: A Contoured Aliphatic Pore Environment in a Cubic Framework with Potential for Benzene Separation Applications. *J. Am. Chem. Soc.* **2019**, *141*, 3828–3832.

[23] Macreadie, L. K.; Babarao, R.; Setter, C. J.; Lee, S. J.; Qazvini, O. T.; Seeber, A. J.; Tsanaktsidis, J.; Telfer, S. G.; Batten, S. R.; Hill, M. R. Enhancing Multicomponent Metal–Organic Frameworks for Low Pressure Liquid Organic Hydrogen Carrier Separations. *Angew. Chem. Weinheim Bergstr. Ger.* **2020**, *132*, 6146–6154.

[24] Shimomura, S.; Horike, S.; Matsuda, R.; Kitagawa, S. Guest-Specific Function of a Flexible Undulating Channel in a 7,7,8,8-Tetracyano-p-Quinodimethane Dimer-Based Porous Coordination Polymer. *J. Am. Chem. Soc.* **2007**, *129*, 10990–10991.

[25] Zhang, J.-P.; Chen, X.-M. Exceptional Framework Flexibility and Sorption Behavior of a Multifunctional Porous Cuprous Triazolate Framework. *J. Am. Chem. Soc.* **2008**, *130*, 6010–6017.

[26] Lin, J.-B.; Zhang, J.-P.; Zhang, W.-X.; Xue, W.; Xue, D.-X.; Chen, X.-M. Porous Manganese(II) 3-(2-Pyridyl)-5-(4-Pyridyl)-1,2,4-Triazolate Frameworks: Rational Self-Assembly, Supramolecular Isomerism, Solid-State Transformation, and Sorption Properties. *Inorg. Chem.* **2009**, *48*, 6652–6660.

[27] Shimomura, S.; Matsuda, R.; Kitagawa, S. Flexibility of Porous Coordination Polymers Strongly Linked to Selective Sorption Mechanism. *Chem. Mater.* **2010**, *22*, 4129–4131.

[28] Ren, G.; Liu, S.; Ma, F.; Wei, F.; Tang, Q.; Yang, Y.; Liang, D.; Li, S.; Chen, Y. A 9-Connected Metal–Organic Framework with Gas Adsorption Properties. *J. Mater. Chem.* **2011**, *21*, 15909.

[29] Mukherjee, S.; Manna, B.; Desai, A. V.; Yin, Y.; Krishna, R.; Babarao, R.; Ghosh, S. K. Harnessing Lewis Acidic Open Metal Sites of Metal–Organic Frameworks: The Foremost Route to Achieve Highly Selective Benzene Sorption over Cyclohexane. *Chem. Commun.* **2016**, *52*, 8215–8218.

[30] Zhang, L.; Yang, W.; Wu, X.-Y.; Lu, C.-Z.; Chen, W.-Z. A Hydrophobic Metal–Organic Framework Based on Cubane-type $[Co_4(\mu_3\text{-}F)_3(\mu_3\text{-}SO_4)]^{3+}$ Clusters for Gas Storage and Adsorption Selectivity of Benzene over Cyclohexane. *Chemistry* **2016**, *22*, 11283–11290.

[31] Wang, G.-D.; Li, Y.-Z.; Shi, W.-J.; Hou, L.; Zhu, Z.; Wang, Y.-Y. A New Honeycomb Metal–Carboxylate-Tetrazolate Framework with Multiple Functions for CO_2 Conversion and Selective Capture of C_2H_2, CO_2 and Benzene. *Inorg. Chem. Front.* **2020**, *7*, 1957–1964.
